# Comprehensive analysis of B cell repopulation in ocrelizumab-treated patients with multiple sclerosis by mass cytometry and proteomics

**DOI:** 10.1016/j.isci.2025.112383

**Published:** 2025-04-08

**Authors:** Meng Wang, Carolin Otto, Camila Fernández Zapata, Adeline Dehlinger, Gerardina Gallaccio, Lisa-Marie Diekmann, Moritz Niederschweiberer, Patrick Schindler, Peter Körtvélyessy, Desiree Kunkel, Friedemann Paul, Klemens Ruprecht, Chotima Böttcher

**Affiliations:** 1 Experimental and Clinical Research Center, a Cooperation between the Max Delbrück Center for Molecular Medicine in the Helmholtz Association and Charité Universitätsmedizin Berlin, Berlin, Germany; 2 Charité – Universitätsmedizin Berlin, Corporate Member of Freie Universität Berlin and Humboldt-Universität zu Berlin, Experimental and Clinical Research Center, Lindenberger Weg 80, Berlin 13125, Germany; 3 Max Delbrück Center for Molecular Medicine in the Helmholtz Association (MDC), Berlin, Germany; 4 Department of Neurology with Experimental Neurology, Charité – Universitätsmedizin Berlin, Corporate Member of Freie Universität Berlin and Humboldt-Universität zu Berlin, Berlin 10117, Germany; 5 Neuroscience Clinical Research Center, Charité – Universitätsmedizin Berlin, Corporate Member of Freie Universität Berlin and Humboldt-Universität zu Berlin, Berlin 10117, Germany; 6 Department of Neurology, Center for ALS and Other Motor Neuron Disorders, Charité - Universitätsmedizin Berlin, Corporate Member of Freie Universität Berlin, Humboldt-Universität zu Berlin, and Berlin Institute of Health, Berlin, Germany; 7 Flow & MassCytometry Core Facility, Berlin Institute of Health at Charité - Universitätsmedizin Berlin, Berlin 13353, Germany

**Keywords:** Treatment, Immunology, Immune response, Proteomics

## Abstract

Ocrelizumab, an anti-CD20 antibody, depletes CD20^+^ B cells, which subsequently repopulate over months. Little is known about changes in other immune cell populations and molecular markers associated with B cell repopulation. Here, we performed a comprehensive characterization of immune cells from ocrelizumab-treated patients with multiple sclerosis (MS) using mass cytometry. About 50% of patients showed naive B cell repopulation after 6 months mainly with a transitional phenotype, whereas CD27^+^ memory B cells only rarely repopulated. This repopulation was associated with a reduction of memory T cells and activated myeloid cells, as well as reduced expression of activation/migration markers in both cell types. A plasma proteomics analysis identified proteins including TNFRSF13C, associated with B cell depletion and repopulation. Plasma levels of neurofilament light-chain protein declined after ocrelizumab treatment was not linked with B cell repopulation. These findings identify potential soluble markers for monitoring of ocrelizumab treatment in MS.

## Highlights

• aCD20 therapy associated with a reduction of memory T cells and activated myeloid cells
• CD20^+^ naive B cells with a transitional phenotype was majority of repopulating B cells
• Plasma TNFRSF13B, TNFRSF13C, TNFRSF9, TNFSF9, and CXCL13 were declined after aCD20 therapy
• Plasma level of TNFRSF13C was strongly associated with B cell repopulation

## Graphical abstract

**Figure:**
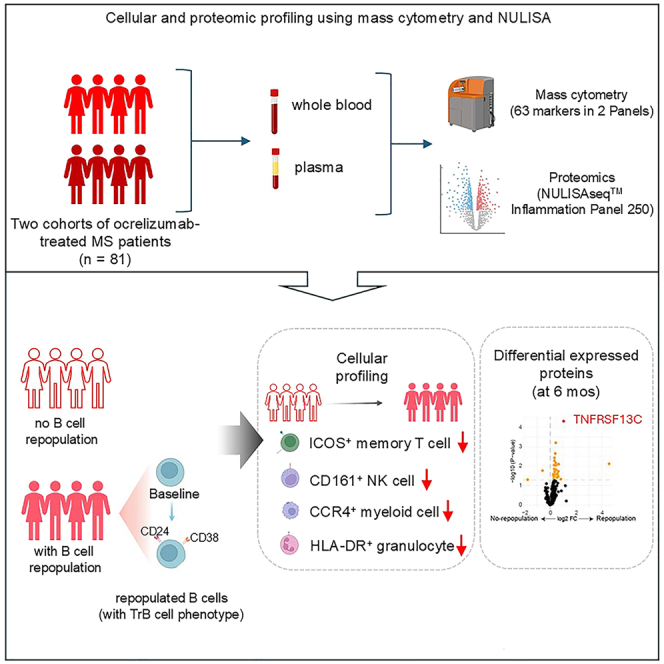

## Introduction

Multiple sclerosis (MS) is a chronic inflammatory demyelinating disease of the central nervous system, which has traditionally been thought to be predominantly mediated by T cells.^1^ However, the highly beneficial effects of B cell depleting anti-CD20 (aCD20) monoclonal antibody therapies in patients with MS have challenged this view and suggest a key role for B cells in the pathophysiology of MS.^2^^,^^3^ Ocrelizumab is a humanized aCD20 monoclonal antibody approved for the treatment of relapsing-remitting MS (RRMS) and primary progressive MS (PPMS).^4^ CD20 is expressed on majority of B cells, from pre-B cells to naive and memory B cells, but not on earlier stage pro-B cells, terminally differentiated plasmablasts, and antibody-producing plasma cells.^5^ Following binding to CD20, ocrelizumab depletes B cells through several different mechanisms, including complement-dependent cytotoxicity and antibody-dependent cellular cytotoxicity.^6^ Due to its selective depletion of CD20^+^ B cells, CD20^−^ B sub-populations are not affected by ocrelizumab treatment.^7^ Apart from depleting CD20-expressing B cells, aCD20 therapies may also influence the phenotype and composition of other immune cells such as CD4^+^ and CD8^+^ T cells, especially their memory compartments.^8^^,^^9^^,^^10^ However, effects of ocrelizumab on non-B cells, including innate immune cells, such as granulocytes, have hitherto not been investigated comprehensively.

B cell repopulation typically occurs at around 6 months following administration of aCD20 therapies.^8^^,^^11^ Repopulating B cells were found to exhibit a transitional phenotype with increased activation profiles.^7^^,^^9^ However, the precise sub- and phenotypes of repopulating B cells and their development in the long-term remain to be assessed. Furthermore, it is also unclear whether B cell repopulation is associated with changes in other immune cell subsets including granulocytes, monocytes, and natural killer (NK) cells. Additionally, little is known about soluble markers potentially correlating with B cell repopulation.

Here, we comprehensively characterized the immune cell repertoire of patients with MS undergoing either short-term or long-term ocrelizumab treatment using high-dimensional mass cytometry (also referred to as cytometry by time-of-flight, CyTOF) with two antibody panels targeting a total of 63 protein markers, identifying at least 72 different immune cell clusters. We put a particular focus on repopulating B cell sub-populations 6 months after treatment and comparatively determined the alterations in non-B cell populations in patients with and without B cell repopulation. Furthermore, using targeted proteomics analysis by NUcleic acid Linked Immuno-Sandwich Assay, NULISA, we identified proteins linked to B cell depletion and/or repopulation. Altogether, our findings provide insights into possible mechanisms of action and identify potential soluble markers for therapy monitoring of anti-CD20 therapies in MS.

## Results

### Patients

**Figure 1:**
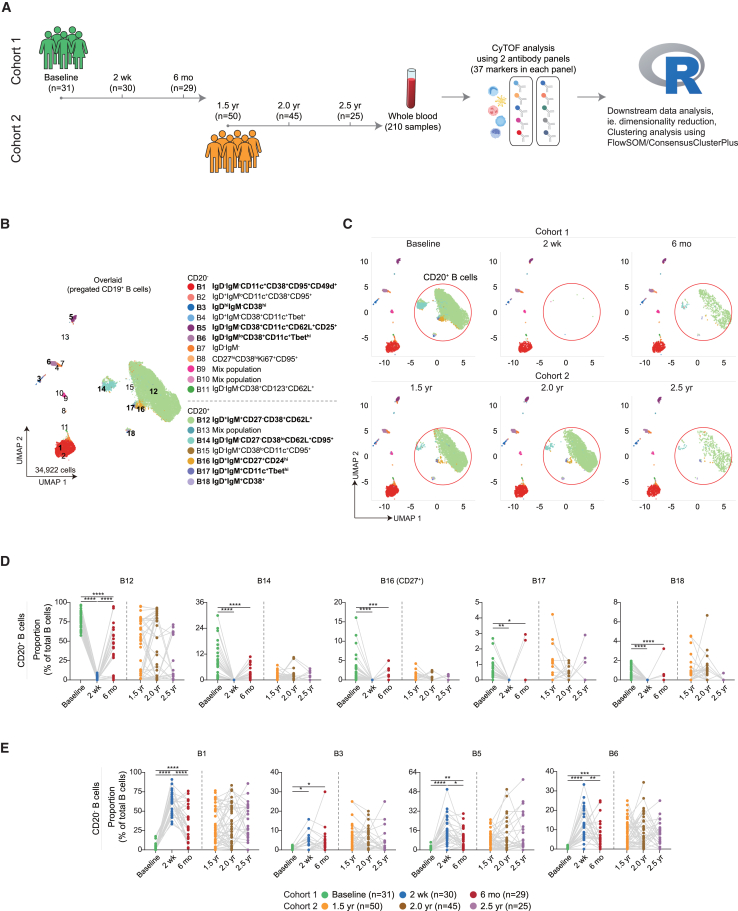
B cell depletion and repopulation after ocrelizumab treatment (A) Schematic overview of two non-overlapping longitudinal cohorts of ocrelizumab-treated patients with MS. In cohort 1, whole blood samples were analyzed at three time points, i.e., before the first ocrelizumab infusion (Baseline, *n* = 31), 2 weeks (2 weeks, *n* = 30) and 6 months (6 months, *n* = 29) after the first infusion. In cohort 2, long-term effects were determined approximately 1.5 (1.5 years, *n* = 50), 2 (2.0 years, *n* = 45), and 2.5 (2.5 years, *n* = 25) years after the first infusion. Whole blood samples were analyzed using streamlined CyTOF analysis workflow. (B) UMAP projection, coloring indicates 1–18 clusters. The phenotype of each cluster is shown based on the median expression of selected markers. (C) UMAP plots showing the depletion of CD20^+^ B cell subpopulations (red circle) at 2 weeks and 6 months as well as 1.5 years, 2.0 years, and 2.5 years after first ocrelizumab infusion. (D and E) Dot plots demonstrating the depletion and repopulation of five CD20^+^ B cell clusters (D) and four CD20^−^ B cell subsets (E) over five time points. Each dot represents the value of one patient. The lines connect longitudinal data points from the same patient. Statistical significance was assessed using a linear mixed model with random effects (Patient_id) and fixed effects (time point). The Bonferroni method was used to control FDR. ∗*p* < 0.05, ∗∗*p* < 0.01, ∗∗∗*p* < 0.001, ∗∗∗∗*p* < 0.0001.

To evaluate effects of ocrelizumab on the immune cell repertoire of patients with MS, we applied single-cell high-dimensional mass cytometry in combination with algorithm-based data analysis to deeply characterize compositional and phenotypic changes of multiple immune cells in two non-overlapping longitudinal MS patient cohorts (Figure 1A). While cohort 1 included samples before start of aCD20 therapy (baseline) as well as 2 weeks and 6 months thereafter, cohort 2 included samples collected 1.5, 2, and 2.5 years after initiation of ocrelizumab, but no baseline samples were available for cohort 2. Demographic and clinical data of both patient cohorts are summarized in Table 1. All patients received ocrelizumab treatment as part of routine therapy. Furthermore, before every infusion, routine blood tests, including serum immunoglobulin measurements and blood cell count, were routinely performed and evaluated by a specialized neurologist.

**Table 1:** Clinical and demographic patient characteristics

	Cohort 1	Cohort 2
Total no.	31	50
Female/Male	14/17	27/23
Age at baseline, mean ± SD (range, years)	36 ± 11 (20–68)	45 ± 11 (23–69)
MS type (no.)		
RRMS	29	40
PPMS	2	10
Time point (patient no.)		
Baseline	31	–
2 weeks	30	–
6 months	29	–
1.5 years	–	50
2.0 years	–	45
2.5 years	–	25
Disease duration, mean ± SD (range)	4.9 ± 7.6 (0–31.3) (years)	8.4 ± 6.9 (1–27) (years)
EDSS at baseline, median (range)	1.5 (0–7.5)	2.5 (0–7)
DMT before OCRE onset		
No	13	15
Yes		
DMF	11	10
GA	8	5
IFN	5	21
TRF	2	4
FTY	3	11
NTZ	2	11
MIT	0	1
ALE	0	2
Relapse before OCRE treatment		
Yes	3	0
No	28	50

RRMS, relapsing remitting multiple sclerosis; PPMS, primary progressive multiple sclerosis; EDSS, expanded disability status scale; DMT, disease-modifying therapy; OCRE, ocrelizumab; DMF, dimethyl fumarate; GA, glatiramer acetate; IFN, interferon; TRF, teriflunomide; FTY, fingolimod; NTZ, natalizumab; MIT, mitoxantrone; ALE, alemtuzumab.

### Compositional changes in major immune cells after ocrelizumab treatment

We first characterized the compositional alterations of major lineage cell subsets, i.e., B cells, T cells, granulocytes, myeloid, and NK cells in whole blood samples using two different CyTOF antibody panels assessing a total of 63 protein markers (Figures S1–S3). On the basis of routine blood test, no differences in absolute cell counts of lymphocytes, neutrophils, and monocytes could be detected at any time points following initiation of aCD20 therapy (Figure S4A). Of note, after treatment, although CD20^+^ B cells were depleted, the total lymphocyte count remained stable across different time points, as B cells represent only a relatively small percentage of all lymphocytes. However, as expected, ocrelizumab treatment led to a strong reduction in the proportion of B cells both at 2 weeks (2 weeks) and at 6 months (6 months) (Figure S4B). Of note, due to the high proportion of granulocytes (>60%) in whole blood samples, the quantified proportions of B and T cells were lower than those previously reported in PBMC samples.^12^ In some patients, B cell repopulation could be detected at 6 months (Figure S4B). Similar findings could also be seen in cohort 2 over 1 year of observation (about 1.5 [1.5 years], 2.0 [2.0 years], and 2.5 years [2.5 years] after first infusion), i.e., the proportion of B cells generally remained low and there was no difference in the proportion of T cells, granulocytes, and myeloid cells, except for a subtle change in NK cells at 2.0 years (Figure S4B). We did not detect significant differences in immune cell composition between patients with different treatment history at baseline (Figure S4C). Next, to gain a deeper understanding of changes within each major cell population and to avoid misinterpretation due to the dynamic changes in cell proportions, we performed pre-gating and sub-clustering analyses of each major immune cell population, i.e., CD19^+^ B cells (Figure S3), CD3^+^ T cells, and CD66b^+^ granulocytes (Figure S2A), as well as myeloid and NK cells (MNK) (Figure S2B).

#### B cells

Sub-clustering analysis of pre-gated CD19^+^ B cells revealed 15 distinct phenotypic B cell sub-clusters and three clusters of mix populations (Figures 1B and S3). Of note, “clusters of mixed populations” refer to a mixture of different cell populations that could not be clearly distinguished due to the limitation of the antibody panels used in this study. At 2 weeks and 6 months, ocrelizumab treatment led to significant reduction in the proportion of four CD27^−^CD20^+^ B cell sub-clusters (cluster B12, B14, B17, and B18) and a CD27^+^ memory B cell subset (B16) as compared to baseline (Figures 1C and 1D). At long-term time points, proportions of B cell subsets were more variable between patients (Figure 1D). Of the nine CD20^−^ B cell sub-clusters, four (i.e., cluster B1, B3, B5, and B6) were proportionally increased after ocrelizumab treatment at 2 weeks and 6 months and remained at high proportions at 1.5 years, 2.0 years, and 2.5 years (Figure 1E), most likely due to strong reduction of CD20^+^ B cell subsets. The other five CD20^−^ sub-populations including CD27^+^CD38^+^ plasma cells (B8) remained unchanged or only subtly changed in proportion over time (Figure S5).

#### T cells and myeloid cells

Concerning effects on sub-clusters of other major lineage cell types, ocrelizumab treatment primarily had early and temporary impacts on T cells, including an increase in CD45RO^+^ memory T cells (i.e., T4, T5, and T10) as well as CD45RO^−^CD127^+^CCR7^+^ naive T cells (T12) and a reduction in CD45RO^−^CD127^−^CCR7^−^ effector CD4/CD8 T cells (i.e., T7 and T9) (Figure S6A). Similar to the T cells, we also observed subtle changes in the proportion of myeloid cells, i.e., CCR4^−^CD33^−^ monocytes (M8 and M12) were decreased at 2 weeks and recovered again at 6 months, whereas CCR4^+^CD33^+^ monocytes (M17) were increased at 2 weeks and decreased at 6 months (Figure S6B). In addition, a decreased proportion of CCR4^+^ DCs (M10) and an increased proportion of CD161^lo^ NK cells (N3) at 6 months could be detected (Figure S6B).

#### Granulocytes

In granulocyte populations, we found that ocrelizumab treatment was associated with an increase in CXCR4^−^HLA-DR^int/+^ granulocytes (i.e., G3, G4 and G8) but a decrease in CXCR4^int/+^ and/or CCR4^+^ granulocytes (i.e., G9 and G17) (Figure S7).

### Cellular and molecular features associated with B depletion and repopulation

**Figure 2:**
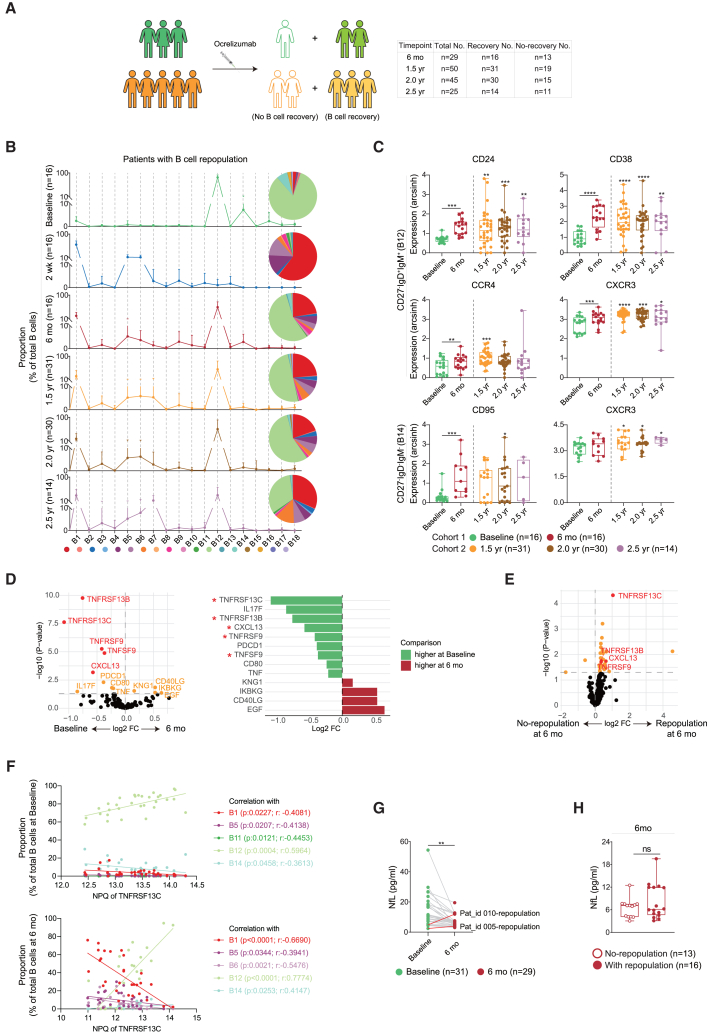
Phenotypic alterations of repopulating B cells after ocrelizumab treatment (A) Schematic overview of patients with and without B cell repopulation following ocrelizumab treatment (6 months: 16 out of 29 [55%]; 1.5 years: 31 out of 50 [62%]; 2.0 years: 30 out of 45 [67%], and 2.5 years: 14 out of 25 [56%]). (B) Histogram and pie charts showing changes in the proportion of 18 B cell clusters in patients with B cell recovery at different time points. Dots indicate mean, and error bars indicate standard error of mean. Coloring indicates 1–18 clusters. (C) Differentially expressed markers (with arcsinh transformation) of repopulating CD27^−^IgD^+^ naive (B12) and CD27^−^IgD^−^ double-negative (B14) B cell clusters at 6 months (*n* = 16), 1.5 years (*n* = 31), 2.0 years (*n* = 30), and 2.5 years (*n* = 14) compared to Baseline (*n* = 16). (D and E) showing significantly differential levels of distinct plasma proteins (analyzed by NULISA technology) between patients at baseline (*n* = 31) and 6 months (*n* = 29) (D) and between patients with and without B cell repopulation at 6 months (E). Volcano plots (left) illustrate the differentially expressed proteins, generated through Limma analysis. Proteins with significant difference after correction for multiple testing (Benjamini & Hochberg) are colored in red; proteins with a significant *p* value but a non-significant adjusted *p* value are shown in yellow, whereas all others are in black. Each dot represents one protein, and horizontal dashed line represents a *p* value threshold of 0.05. Bar plots (right) showing differentially expressed proteins with name. (F) Significant correlation between the proportion of B cell sub-cluster and NPQ of TNFRSF13C at Baseline (upper panel) and 6 months (lower panel). Nonparametric Spearman correlation test (r), two-sided. (G and H) Box plots showing levels of NfL in plasma of patients at baseline (*n* = 31) and 6 months (*n* = 29) (G) and between patients with and without B cell repopulation at 6 months (H). Each dot represents one patient. Statistical significance was determined using Mann-Whitney U tests.

In some patients (at 6 months: 16 out of 29 [55%]; 1.5 years: 31 out of 50 [62%]; 2.0 years: 30 out of 45 [67%]; 2.5 years: 14 out of 25 [56%]), repopulation of CD20^+^ B cells was detected (Figures 1D, 2A, and 2B; Tables S4 and S5). Analysis of the subtype of repopulating B cells showed that a naive B cell population (B12) represented the majority of repopulated B cells 6 months after treatment initiation (Figures 1C and 2B). Interestingly, among the 16 patients with B cell repopulation at 6 months, only 4 (25%) showed repopulation of CD27^+^ memory B cells (B16) (Table S4). In line with previous studies,^7^^,^^9^ the repopulated CD20^+^IgD^+^IgM^+^ mature naive B cells (B12) showed a shift toward transitional phenotypes, with increased expression of CD24 and CD38 at 6 months to 2.5 years as compared with baseline, as well as an increased expression of CXCR3 and CCR4 (Figure 2C, upper panel). Furthermore, we also detected an increase in the expression of CD95 and CXCR3 on a repopulated CD20^+^IgD^−^IgM^−^CD27^−^CD95^+^ B cell cluster (B14) (Figure 2C, lower panel).

To identify soluble mediators linked to B cell depletion and repopulation, we performed a proximity extension assay of plasma samples collected at baseline and 6 months after treatment using an NULISA panel targeting 250 protein markers involved in inflammatory processes. Limma analysis identified significant decreases (adjusted *p*-value <0.05) of five markers (i.e., TNFRSF13B, TNFRSF13C, TNFRSF9, TNFSF9, and CXCL13) at 6 months (Figure 2D). While some of these markers, i.e., TNFRSF13B, TNFRSF9, CXCL13, and TNFRSF13C, were found at higher levels in the plasma of patients with B cell repopulation at 6 months, only TNFRSF13C was significantly associated with repopulating B cells (Figure 2E). Furthermore, TNFRSF13C levels in plasma positively correlated with the proportion of the naive B12 cluster at baseline and even more so with the proportion of repopulating B12 cells at 6 months (Figure 2F). In contrast, proportions of the other B cell clusters showed either no (data not shown) or negative correlations with levels of TNFRSF13C in plasma at baseline and 6 months (Figure 2F).

Following initiation of ocrelizumab therapy, immunoglobulin M (IgM), but not IgG, levels decreased at 6 months compared to baseline (Figures S8A and S8B). MS patients with B cell recovery exhibited both higher IgG and IgM levels at 2.5 years compared with those without B cell recovery (Figures S8C and S8D).

### No association of B cell repopulation with plasma neurofilament light-chain protein levels

Neurofilament light-chain protein (NfL) is a marker for neuroaxonal injury, which was previously shown to decrease following initiation of anti-CD20 therapy in patients with MS.^13^ In line with these findings, we observed reduced NfL plasma levels at 6 months after treatment initiation compared to baseline in our patients (cohort 1) as well (Figure 2G). However, there were two patients who showed an NfL increase at 6 months, both of whom also had repopulated B cells at 6 months (Figure 2G). Nevertheless, there was overall no significant difference in plasma NfL levels between patients with and without B cell repopulation at 6 months (Figure 2H).

### B cell recovery associated with less memory T cells and reduced proinflammatory myeloid cells

**Figure 3:**
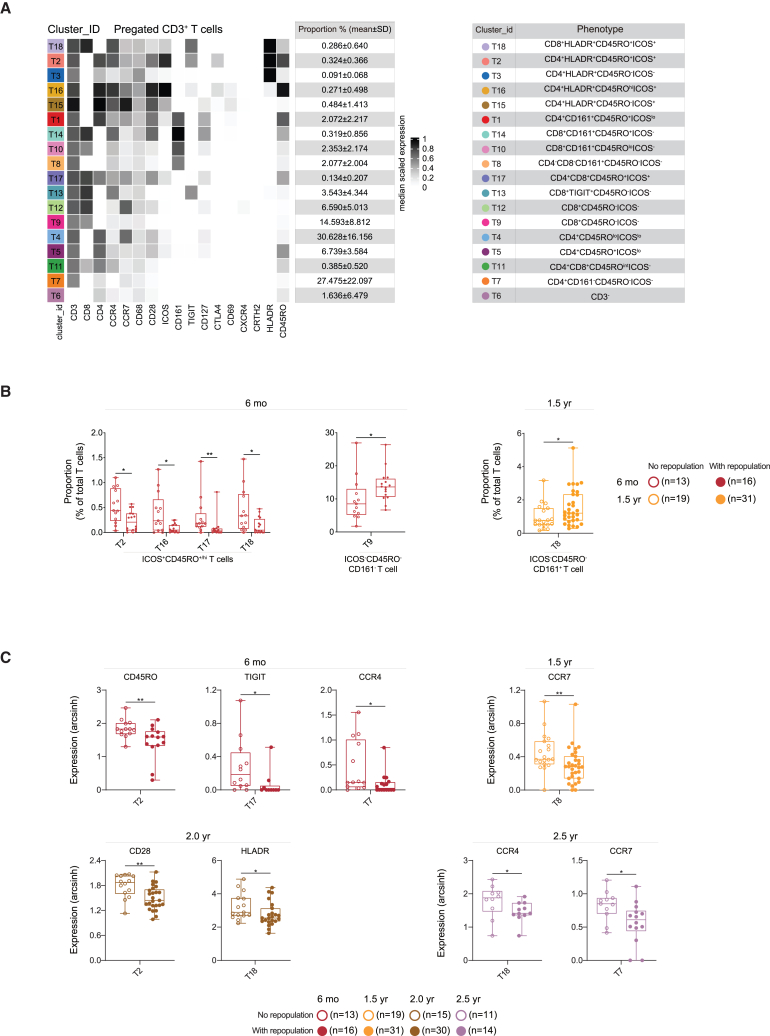
B cell repopulation associates with compositional and phenotypic changes of T cell subsets (A) Phenotypic heatmap and tables of 18 defined cluster (left panel) identities depicting the proportion and median expression levels of selected markers for CD3^+^ T cells in (A). Heat colors of expression levels have been scaled for each marker individually (to the 1st and 5th quintiles) (black, high expression; white, no expression). (B) Differences in proportional changes of T cell sub-clusters in patients with and without B cell recovery at different time points, as compared with those without recovery. (C) Boxplots showing differences in marker expression of T cell subpopulations between patients with and without B cell recovery at different time points. Each dot represents one patient. Whisker plots show the min (smallest) and max (largest) values. The line in the box denotes the median. Statistical significance was determined using a two-stage step-up method of Benjamini, Krieger, and Yekutieli correction for cluster proportion (B) and Mann-Whitney U test for marker expression (C). ∗*p* < 0.05, ∗∗*p* < 0.01.

We next compared non-B cell profiles in patients with and without B cell repopulation at all time points. At 6 months, patients with B cell repopulation displayed lower proportions of CD45RO^+^ICOS^+^ memory T cell clusters (T2, T16, T17, and T18) but higher proportions of CD45RO^−^ICOS^−^CCR7^lo/−^CD127^−^ effector T cell clusters (T9 at 6 months and T8 at 1.5 years) (Figures 3A and 3B). Furthermore, the expression levels of CCR7, TIGIT, CD45RO, CD28, HLADR, and CCR4 were lower in memory T cells in patients with B cell repopulation (Figure 3C), suggesting decreased pro-inflammatory and/or memory phenotypes of T cells in these patients.

**Figure 4:**
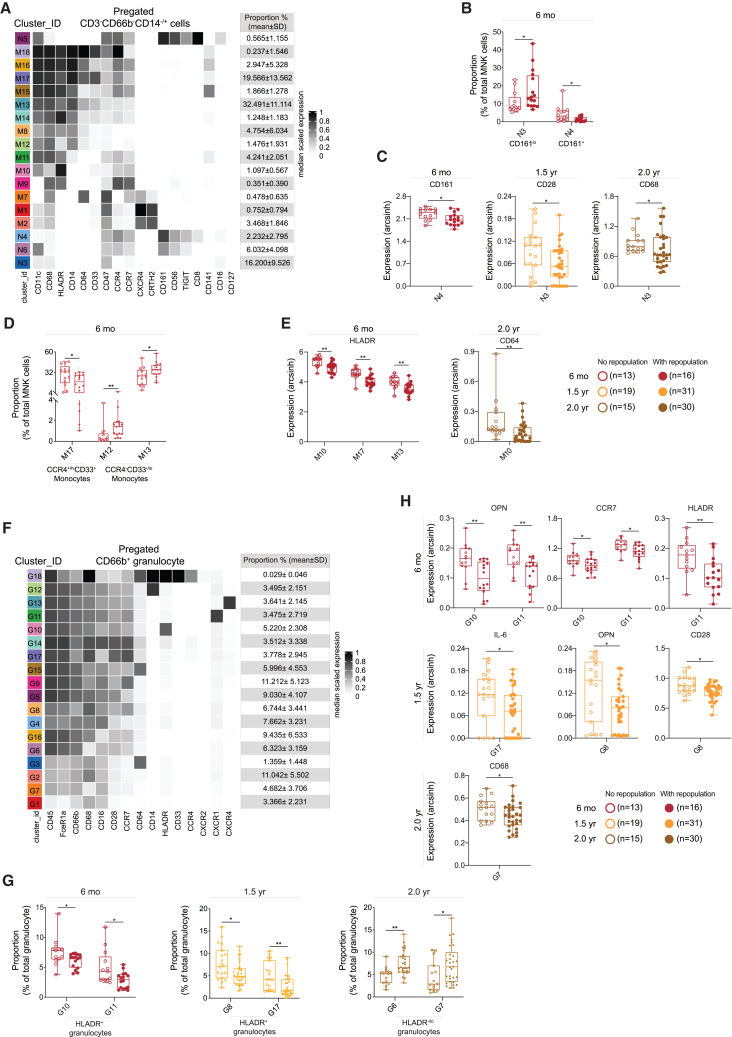
B cell repopulation associated with compositional and phenotypic changes in MNK cell and granulocyte subsets (A) Phenotypic heatmap of cluster identities depicting the median expression levels of selected markers for MNK cells. Heat colors of expression levels have been scaled for each marker individually (to the 1st and 5th quintiles) (black, high expression; white, no expression). (B) Boxplots showing differentially abundant NK cell clusters in patients with and without B cell repopulation at different time points as compared with patients without B cell repopulation. (C) Boxplots showing significant differences in marker expression of NK cell subpopulations between patients with and without B cell recovery at different time points. (D) Proportional differences in myeloid cell proportions between patients with and without B cell recovery at different time points. (E) Boxplots showing altered marker expression of myeloid cell subpopulations in patients with and without B cell repopulation. (F) Phenotypic heatmap of cluster identities depicting the median expression levels of selected markers for granulocytes. (G) Differences in proportion of granulocyte clusters in patients with B cell recovery at different time points as compared with patients without B cell repopulation. (H) Boxplots showing differences in marker expression of granulocyte subpopulations between patients with and without B cell recovery at different time points. Each dot represents one patient. Whisker plots show the min (smallest) and max (largest) values. The line in the box denotes the median. Statistical significance was determined using two-stage step-up method of Benjamini, Krieger, and Yekutieli correction for cluster proportion (B, D, and G) and Mann-Whitney U test for marker expression (C, E, and H). ∗*p* < 0.05 and ∗∗*p* < 0.01.

Similarly, we also found lower proportions of CD161^+^ proinflammatory NK cells (N4 at 6 months)^14^ as well as lower CD161 expression level in this subset of patients with B cell repopulation, whereas CD161^lo^ NK cells (N3 at 6 months) were found at higher proportion in these patients as compared with those of patients without B cell repopulation (Figures 4A–4C). In CD161^lo^ NK cells (N3), we also detected lower level of CD28 and CD68 expression in patients with B cell repopulation (Figure 4C).

In patients with B cell repopulation, we also detected a lower proportion of CCR4^+^CD141^lo/−^ myeloid cells (M17 at 6 months),^15^ whereas CCR4^−^ myeloid cells (M12 and M13 at 6 months) were found at higher proportion as compared with those of patients without repopulation (Figure 4D). The expression levels of HLA-DR and CD64 were decreased in several myeloid cell subsets of patients with B cell repopulation (Figure 4E), as compared with the expression levels in patients without B cell repopulation.

Interestingly, a lower proportion of HLA-DR-expressing granulocytes was detected in patients with B cell repopulation (i.e., at 6 months [G10 and G11] and 1.5 years [G8 and G17]), whereas HLA-DR^−/lo^ granulocytes were present at a higher proportion (i.e., at 2.0 years [G6 and G7]; Figures 4F and 4G). In association with B cell repopulation, a small reduction of osteopontin (OPN), CCR7, interleukin-6 (IL-6), CD28, and CD68 was detected in diverse subsets of granulocytes (mainly at 6 months, 1.5 years, and 2.0 years) (Figure 4H).

Taken together, we detected decreased memory and/or activation phenotypes in T and myeloid cells in association with B cell repopulation at 6 months, showing that B cell depletion by anti-CD20 therapies is associated with subtle changes also in non-B cell compartments.

## Discussion

The key findings of this detailed and comprehensive study of immune cell profiles and soluble markers in aCD20-treated patients with MS are the following: (1) aCD20 therapy depleted four CD27^−^CD20^+^ B cell sub-clusters (cluster B12, B14, B17, and B18) and a CD27^+^ memory B cell subset (B16) at 2 weeks after treatment start; (2) aCD20 treatment likewise led to early but more subtle compositional changes of T cells, monocytes, dendritic cells, NK cells, and granulocytes; (3) 6 months after treatment initiation, B cell repopulation was detectable in about 50% of patients; the major repopulating B cell sub-cluster were CD20^+^IgD^+^IgM^+^ mature naive B cells (B12) with a transitional phenotype, whereas CD27^+^ memory B cells (B16) repopulated only infrequently; (4) plasma levels of five soluble markers (i.e., TNFRSF13B, TNFRSF13C, TNFRSF9, TNFSF9, and CXCL13) declined at 6 months after start of aCD20 therapy, with TNFRSF13C being most strongly associated with B cell repopulation; (5) B cell repopulation was associated with slightly higher long-term serum immunoglobulin levels, but not with plasma NfL levels at 6 months; and (6) B cell repopulation at 6 months was associated with less memory T cells and reduced proinflammatory myeloid cells.

The rapid depletion of CD20^+^, but not CD20^−^, B cells following initiation of aCD20 therapy was an expected finding consistent with numerous previous observations.^4^^,^^5^^,^^6^^,^^7^ However, the present high-dimensional mass cytometry approach allowed to identify four CD27^−^CD20^+^ naive and one CD27^+^CD20^+^ memory B cell sub-clusters among CD20^+^ B cells depleted by ocrelizumab, which may be considered the primary targets of aCD20 therapies. Given the highly beneficial effects of B cell depletion by aCD20 therapies in MS, it is tempting to speculate that one or more of the five B cell clusters identified herein may be key drivers of the disease process of MS. Notably, strong evidence suggests a causative role of the Epstein-Barr virus (EBV) in MS.^16^^,^^17^^,^^18^ Although the underlying mechanisms currently unclear, it is firmly established that the site of EBV persistence in humans are memory B cells. It was therefore proposed that the effects of aCD20 therapies in MS may be due to depletion of circulating memory B cells harboring EBV.^19^ Although further work will be required to verify or falsify this hypothesis, our current findings show that circulating CD27^+^CD20^+^ memory B cells are practically completely depleted 2 weeks after the start of aCD20 therapy.

At present, it remains unclear whether the subtle changes in T cell, monocyte, dendritic cells, NK cell, and granulocyte sub-clusters occurring after initiation of aCD20 therapy may contribute to the beneficial effect of aCD20 therapies in MS. Still, our findings underscore how the targeted depletion of one immune cell subset may be associated with subtle repercussions in various other immune cell subsets.

The detection of reappearing B cells in some of the patients at 6 months after the last aCD20 infusion is overall consistent with data from the pivotal clinical trials of a CD20 therapies in MS.^4^^,^^20^ The transitional regulatory phenotype (i.e., CD24^+^CD38^+^) of the majority of the repopulated B cells is in line with previous studies.^9^^,^^11^ In addition to the recurrence of CD24^+^CD38^+^ transitional regulatory B cells, we also detected a significant repopulation of a IgD^−^CD27^−^ double-negative (DN) B cell subset (B14). DN B cells have been involved in different immune-mediated diseases including auto-immune and chronic inflammatory diseases.^21^ This rare population is possibly heterogeneous and may have various functions, e.g., an immunosuppressive role in tumor environment or contributing to autoimmunity.^22^ The repopulated B14 B cell subset exhibited an increased expression of CD95, a cell surface receptor belonging to the tumor necrosis factor (TNF) family. CD95 has long been recognized as a death signal that plays a crucial role in maintaining immune tolerance and homeostasis.^23^ It is expressed on the surface of various immune cells, including B cells.^24^ In pathological conditions, CD95 can activate a signaling pathway that leads to apoptosis, facilitating the elimination of non-specific and autoreactive B cells,^25^ thereby potentially serving a regulatory function. Given that the repopulating DN B cells demonstrated heightened CD95 expression, it is plausible that they may enhance their regulatory phenotype.

Evidence obtained from clinical trials of various immunotherapies for MS suggests memory B cells as a central player in the development of MS.^26^ Indeed, practically all immunotherapies beneficial for MS diminish the number of circulating memory B cells and/or interfere with their function. In contrast, drugs that augment B memory cell function (e.g., atacicept and infliximab) worsen MS.^26^ Herein, we found that repopulation of IgM^+^IgD^+^CD27^+^ memory B cells in MS patients following treatment with ocrelizumab occurs less frequently than repopulation of naive B cells. IgM^+^IgD^+^CD27^+^ memory B cells, long-living cells with a similar longevity as class-switched memory B cells,^27^ have been demonstrated *in vitro* in numerous stimulation conditions to be easier to activate than naive B cells.^28^^,^^29^ Protracted depletion of CD27^+^ memory B cells may thus be one reason for the long-lasting effects of ocrelizumab and might also explain why extending infusion intervals is usually not associated with disease worsening,^30^^,^^31^ albeit B cell repopulation.

At 6 months after treatment, a targeted proteomic analysis using NULISA revealed a significant reduction in five proteins, i.e., TNFRSF13B, TNFRSF13C, TNFRSF9, TNFSF9, and CXCL13, which are known to be involved in B cell functions and B-T cell activation.^32^^,^^33^ Especially, the reduction of CXCL13, a crucial B cell chemoattractant, implies reduced B cell trafficking to inflammatory sites, potentially contributing to the therapeutic effect of ocrelizumab.^34^ Among the identified proteins, TNFRSF13C was strongly associated with CD20^+^IgD^+^IgM^+^ mature naive B cells (B12), suggesting a potential use of TNFRSF13C as a soluble marker for monitoring this B cell sub-cluster as a surrogate for treatment responses to aCD20 therapies.^32^^,^^35^ However, future studies will be required for a comprehensive validation of TNFRSF13C as a treatment response marker for aCD20 therapies.

Our study confirmed a reduction in plasma levels of NfL after treatment initiation with ocrelizumab, demonstrating the efficacy of ocrelizumab in mitigating neuroaxonal damage as early as 6 months post-treatment.^13^ A cross-sectional comparison demonstrated no significant differences in plasma levels of NfL between patients with and without B cell repopulation at 6 months after ocrelizumab treatment, suggesting that B cell repopulation is not associated with neuroaxonal damage at this early time point. Nevertheless, the only two patients with an increase in plasma NfL levels at 6 months also showed B cell repopulation at 6 months. Altogether, larger studies with longer follow-up periods will be necessary to comprehensively assess the potential impact of B cell repopulation on neuroaxonal damage in MS.

Patients with B cell repopulation displayed slightly higher levels of serum IgG and IgM in the long term compared to those without repopulation, consistent with the concept that the more B cells repopulate, the more antibody secreting cells may subsequently be generated.

In addition to the phenotypic changes in repopulating B cells, we also observed phenotypic changes in several other immune cell types. Patients with B cell repopulation presented a smaller proportion of multiple ICOS^+^CD45RO^+/hi^ memory T cells (i.e., both CD4^+^ and CD8^+^ T cells), whereas CCR7^lo^ T cells became increased in proportion, as compared with patients without B cell repopulation. In postmortem white matter of patients with MS, CD4^+^ memory T cells show positive correlations with antibody-secreting B cells, which may be due to local interplay between these two cell populations in active white-matter lesions.^36^ Furthermore, B cell repopulation was also associated with decreased migration and activation markers on T cells, e.g., HLA-DR, CCR7, and CCR4. Altogether, these results may suggest that B cell repopulation with a transitional phenotype may lead to a shift in the T cell compartment toward less activated T cell states as well as T cell-B cell interaction. Whether this shift might play a role in pathogenesis of MS has to be further investigated.

Innate immunity is the first line of host defense against invading pathogens. In our study, we characterized the heterogeneity of granulocytes, a key component of innate immunity, and demonstrated that depletion of CD20^+^ B cells led to a subtle change in granulocyte and myeloid cell composition and phenotypes. At 2 weeks after the first ocrelizumab infusion, we detected short-term changes in the proportion of CD14^+^ monocytes and CCR4^+^ DCs, which were normalized to the baseline at later time points. In the granulocyte population, proportional and phenotypic changes were mainly detected at later time points (1.5 years–2.5 years), i.e., an increase in the proportion of HLA-DR^int/+^ population. Similar to changes in T cell populations, a reduction of CD161^+^ cytotoxic NK cells and CCR4^+^ monocytes was identified (along with increased CD161^lo^CD47^+^ NK cells and CCR4^−^ monocytes) in patients with B cell repopulation. CCR4, a key player in regulating lymphocytes involved in inflammation, can be also expressed by monocytes^37^^,^^38^ and contributes to the pathogenesis of experimental autoimmune encephalomyelitis and MS.^15^^,^^39^^,^^40^ Furthermore, the regulation of HLA-DR and CD64 expression on myeloid cells, in particular monocytes, has emerged as an important mechanism of responses to inflammation or induced immunosuppression,^41^^,^^42^ thus suggesting that B cell repopulation is associated with reduced pro-inflammatory responses of monocytes. In a previous study,^43^ we demonstrated that HLA-DR^lo/+^ granulocyte sub-populations are positively correlated with humoral and cell-mediated immunity in the context of vaccination. In this study, we also detected a significant reduction of the proportion of HLA-DR^+^ granulocytes associated with B cell recurrence, whereas the proportion of HLA-DR^−/lo^ granulocytes was found proportionally increased, suggesting decreased activation of granulocytes following B cell repopulation. Furthermore, B cell repopulation following ocrelizumab treatment was also associated with a reduction of HLA-DR and CCR7 expression on multiple cell types including T cells and granulocytes. CCR7 is known as a receptor critically involved in the migration of myeloid cells to T cell zones in lymph nodes, initiating distinct immune response and potentially contributing to many inflammatory diseases, including MS.^44^^,^^45^^,^^46^^,^^47^ In addition, one study in mice has shown that interferon β (IFN-β) treatment could inhibit the expression of CCR7, potentially contributing to the therapeutic effect.^48^

In conclusion, we conducted a comprehensive immune profiling in whole blood of ocrelizumab-treated patients with MS at the single-cell level. This identified B cell subclusters primarily targeted by aCD20 therapies and permitted a detailed characterization of repopulating B cell as well as associated changes in non-B immune cell populations. We also identified soluble markers associated with B cell depletion and repopulation. Overall, our results provide valuable insights into the effects of ocrelizumab on the immune landscape at the cellular and proteomic level in patients with MS, emphasizing a long-lasting impact on CD27^+^ memory B cells, alterations in non-B immune cell subsets linked to B cell repopulation, and changes in key immune-regulatory proteins.

### Limitations of the study

A limitation of our study is the lack of a baseline sample from participants of cohort 2, which only included samples obtained at 1.5, 2.0, and 2.5 years post-initiation of ocrelizumab treatment. We recognize that utilizing baseline data from cohort 1 as a reference for cohort 2 is not a perfect substitute for the missing baseline data of cohort 2. However, the primary objective of cohort 2 was to cross-sectionally investigate sustained effects of ocrelizumab therapy on various immune cell populations, with a particular emphasis on B cell repopulation. Given the sufficiently large patient sample at baseline, we were able to conduct a robust cross-sectional comparison of B cell phenotypes (shown in Figure 2) in cohort 1 and cohort 2. This analysis revealed consistent changes in the phenotype of repopulated B cells, specifically a shift toward regulatory phenotypes. Furthermore, in terms of statistical analyses conducted in this study, we performed cross-sectional comparisons between patients with and without B cell repopulation at each time point, thereby rendering baseline data less critical for these specific evaluations.

The CyTOF methodology applied in this work has certain technical limitations. While CyTOF enables the unambiguous and simultaneous characterization of immune phenotypes across diverse cell populations, including granulocytes, in whole blood, it employs an antibody-based approach to assess single-cell phenotypes. This method is not entirely unbiased, which may lead to the exclusion of certain relevant immune cell subsets (e.g., CXCR5-expressing cells that may be correlated to the change of plasma CXCL13 level) due to the characteristics of the antibody panels used. Consequently, further investigations are necessary to explore novel markers, particularly those that assess specific T cell populations, potentially identified through alternative single-cell technologies. Additionally, the workflow of CyTOF employed in this study—including barcoding and sample pooling—prevented us from calculating the absolute cell counts for each major population. However, our analysis of proportional changes enabled us to detect significant shifts in the composition of sub-populations within broader cell types, e.g., B cells, T cells, granulocytes, myeloid, and NK cells.

Our study was not designed to and could not address the relationship between the depth of B cell depletion or the extent of B cell repopulation and clinical outcomes such as disease activity or progression. Indeed, following the initiation of ocrelizumab therapy, the majority of patients with MS exhibits only minimal clinical and radiographic disease activity, and any differences in disease progression will only become apparent over longer time periods. Much larger and longer studies will be required to investigate potential association of the immunological phenotypes identified in this work with clinical outcomes.

Finally, the biological functions and pathogenic consequences of the identified changes in immune cell subsets, including repopulating B cells, as well as in B-cell-associated soluble markers warrant further investigation.

## Resource availability

### Lead contact

Further information should be directed and will be fulfilled by the lead contact, Dr. Chotima Böttcher (chotima.boettcher@charite.de).

### Materials availability

This study did not generate new unique reagents or materials.

### Data and code availability

• Data are available as supplementary tables or from the lead contact upon request.
• Codes used for CyTOF data analysis in this study were previously published by Crowell H et al. 2022 and available on https://github.com [https://github.com/HelenaLC/CATALYST].
• Any additional information required to reanalyze the data reported in this working paper is available from the lead contact upon request.

## Acknowledgments

We would like to acknowledge the assistance of the BIH Cytometry Core Facility. M.W. is recipient of a PhD scholarship from the Chinese Scholarship Council (CSC). C.B. was funded by the 10.13039/501100001659Deutsche Forschungsgemeinschaft (DFG, the German Research Foundation—Project-ID 259373024—CRC/TRR 167 [B05] and the Federal Ministry of Education and Research (BMBF—TahRget [SP3]).

## Author contributions

C.B., C.O., K.R., and F.P. conceived and designed the project. C.B., C.F.Z., and D.K. designed the antibody panels for mass cytometry. C.O., M.N., and P.S. recruited the patients and provided the patients’ clinical data. M.W., C.F.Z., A.D., and G.G. performed CyTOF experiments and data analyses. M.W. and L.-M.D. analyzed the NULISA data. P.K. analyzed the plasma NfL data. C.B., K.R., M.W., and C.O. analyzed and interpreted the data. C.B., K.R., F.P., M.W., C.O., C.F.Z., A.D., G.G., M.N., and P.S. wrote the manuscript.

## Declaration of interests

F.P. received research support from F. Hoffmann-La Roche Ltd., Alexion Pharma Germany GmbH and Horizon Therapeutics Ireland DAC. K.R. received research support from Novartis, Merck Serono, German Ministry of Education and Research, European Union (821283-2), Stiftung Charité, Guthy-Jackson Charitable Foundation, and Arthur Arnstein Foundation; received travel grants from Guthy-Jackson Charitable Foundation; received speaker’s honoraria from Virion Serion and Novartis; was a participant in the BIH Clinical Fellow Program funded by Stiftung Charité.

## STAR★Methods

### Key resources table

**Table:** 

REAGENT or RESOURCE	SOURCE	IDENTIFIER
**Antibodies**		

CCR4	Standard BioTools	clone L291H4; Cat# 3158032A; RRID: AB_2893003
CCR7	Standard BioTools	clone G043H7; Cat# 3167009A; RRID: AB_2858236
CD11b	Standard BioTools	clone ICRF44; Cat# 3209003B; RRID: AB_2687654
CD11c	Biolegend	clone But5; Cat# 337221; RRID: AB_2562834
CD123	Biolegend	clone 6H6; Cat# 306002; RRID: AB_314576
CD127	Standard BioTools	clone A019D5; Cat# 3176004B; RRID: AB_3665122
CD138	Standard BioTools	clone DL-101; Cat# 3145003B; RRID: AB_3677805
CD14	Standard BioTools	clone RM052; Cat# 3160006B; RRID: AB_2661801
CD141	Standard BioTools	clone M80; Cat# 3166017B; RRID: AB_2892693
CD16	Standard BioTools	clone 3G8; Cat# 3148004B; RRID: AB_3665424
CD161	Biolegend	clone HP-3G10; Cat# 339919; RRID: AB_2562836
CD19	Standard BioTools	clone HIB19; Cat# 3142001B; RRID: AB_3661857
CD1c	Biolegend	clone L161; Cat# 331502; RRID: AB_1088995
CD20	Standard BioTools	clone 2H7; Cat# 3171012B; RRID: AB_2802112
CD206	Biolegend	clone 15-2; Cat# 3168008B; RRID: AB_2661805
CD24	Standard BioTools	clone ML5; Cat# 3169004B; RRID: AB_2688021
CD25	Standard BioTools	clone 2A3; Cat# 3149010B; RRID: AB_2756416
CD27	Standard BioTools	clone O323; Cat# 3167002B; RRID: AB_3094744
CD28	BD Bioscience	clone L293; Cat# 340975; RRID: AB_400197
CD3	Standard BioTools	clone UCHT1; Cat# 3154003B; RRID: AB_2811086
CD33	Standard BioTools	clone WM53; Cat# 3169010B; RRID: AB_2802111
CD34	Standard BioTools	clone 581; Cat# 3166012B; RRID: AB_2756424
CD38	Standard BioTools	clone HIT2; Cat# 3144014B; RRID: AB_2687640
CD4	Standard BioTools	clone RPA-T4; Cat# 3145001B; RRID: AB_3661845
CD45	Standard BioTools	clone HI30; Cat# 3089003B; RRID: AB_2938863
CD45RO	Standard BioTools	clone UCHL1; Cat# 3165011B; RRID: AB_2756423
CD47	Standard BioTools	clone CC2C6; Cat# 3209004B; RRID: AB_3678049
CD49d	Standard BioTools	clone 9F10; Cat# 3141004B; RRID: AB_2892684
CD56	Standard BioTools	clone NCAM16.2; Cat# 3149021B; RRID: AB_2938638
CD62L	Standard BioTools	clone DREG-56; Cat# 3153004B; RRID: AB_2810245
CD64	Standard BioTools	clone 10.1; Cat# 3146006B; RRID: AB_2661790
CD66b	Standard BioTools	clone 8OH3; Cat# 3152011B; RRID: AB_2661795
CD68	Biolegend	clone Y1/82A; Cat# 333802; RRID: AB_1089058
CD69	Standard BioTools	clone FN50; Cat# 3144018B; RRID: AB_2687849
CD8a	Standard BioTools	clone RPA-T8; Cat# 3162015B; RRID: AB_2811089
CD95	Standard BioTools	clone DX2; Cat# 3164008B; RRID: AB_2858235
CHI3L	abcam	clone EPR19078-157; Cat# ab255864; RRID: AB_2927474
cPARP	Standard BioTools	clone F21-852; Cat# 3143011A; RRID: AB_2927562
CTLA-4	Standard BioTools	clone 14D3; Cat# 3161004B; RRID: AB_2687649
CXCR1	Standard BioTools	clone 8F1/CXCR1; Cat# 3142009B; RRID: AB_3661726
CXCR2	Standard BioTools	clone 5E8/CXCR2; Cat# 3147010B; RRID: AB_3677820
CXCR3	Standard BioTools	clone G025H7; Cat# 3163004B; RRID: AB_2810969
CXCR4	Standard BioTools	clone 12G5; Cat# 3173001B; RRID: AB_3106963
FceR1a	eBioscience	clone AER-37 (CRA1); Cat# 13-5899-82; RRID: AB_466786
HLA-DR	Standard BioTools	clone L243; Cat# 3143013B; RRID: AB_3661844
ICOS	Standard BioTools	clone C398.4A; Cat# 3151020B; RRID: AB_3677860
IgA	Standard BioTools	clone polyclonal; Cat# 3148007B; RRID: AB_2810852
IgD	Standard BioTools	clone IA6-2; Cat# 3146005B; RRID: AB_2811082
IgGK	Standard BioTools	clone MHK-49; Cat# 3160005B; RRID: AB_2810855
IgGL	Standard BioTools	clone MHL-38; Cat# 3151004B; RRID: AB_2810853
IgM	Standard BioTools	clone MHM-88; Cat# 3172004B; RRID: AB_2810858
IL-1β	eBioscience	clone CRM56; Cat# 14-7018-85; RRID: AB_468401
IL-6	Standard BioTools	clone MQ2-13AS; Cat# 3156011B; RRID: AB_2810973
Ki67	Standard BioTools	clone B56; Cat# 3168007B; RRID: AB_2800467
KLRG1	Biolegend	clone 14C2A07; Cat# 368602; RRID: AB_2566256
LAG-3	Standard BioTools	clone 11C3C65; Cat# 3165037B; RRID: AB_2810971
MIP-1β/CCL4	Standard BioTools	clone D211351; Cat# 3150004B; RRID: AB_3677842
OPN	LSBio	clone polyclonal; Cat# C99283; RRID: AB_2194984
PD-1	Standard BioTools	clone EH12.2H7; Cat# 3174020B; RRID: AB_2868402
Tbet	Biolegend	clone 4B10; Cat# 644825; RRID: AB_2563788
TIGIT	Standard BioTools	clone MBSA43; Cat# 3159038B; RRID: AB_3676413
TNF	DVS Sciences	clone MAb11; Cat# 3175023B; RRID: AB_3678032

**Biological samples**		

Whole blood	This paper	N/A
Serum	This paper	N/A
Plasma	This paper	N/A

**Chemicals, peptides, and recombinant proteins**		

Proteomic Stabilizer	SmartTube Inc.	Cat# PROT1-1L
Thaw/Lyse buffer	Smart Tube Inc.	Cat# 501351696
Maxpar® Cell staining buffer	Standard BioTools	Cat# 201068
Methanol-free formaldehyde solution	Fisher Scientific	Cat# 10751395
Permeabilization buffer	Thermo Fisher Scientific	00-8333-56
Iridium intercalator solution	Standard BioTools	201192A

**Critical commercial assays**		

Cell-ID 20-plex Pd Barcoding Kit	Standard BioTools	Cat# 201060

**Software and algorithms**		

FlowJo	FlowJo	Version 10.8.1
R	The R Project for Statistical Computing	Version 4.2.2
GraphPad Prism	GraphPad	Version 8.0.2
SPSS	The IBM SPSS Statistics	Version 24
CATALYST	https://github.com/HelenaLC/CATALYST	1.22.0
FlowSOM	FlowSOM package in R	2.6.0
ConsensusClusterPlus	ConsensusClusterPlus package in R	1.62.0

### Experimental model and study participant details

#### Patient samples

To evaluate short- and long-term effects of ocrelizumab on the immune cell repertoire of patients with MS, we applied single-cell high-dimensional CyTOF technology in a combination with algorithm-based data analysis to deeply characterize compositional and phenotypic changes of immune cells in two non-overlapping longitudinal patient cohorts. All patients were recruited at the MS outpatient clinic of the Department of Neurology, Charité – Universitätsmedizin Berlin, Berlin, Germany. Inclusion criteria were age >18 years, a diagnosis of RRMS or PPMS according to the McDonald criteria of 2017^49^ and treatment with ocrelizumab as part of routine medical treatment.

In cohort 1, treatment with ocrelizumab was initiated during the study period by administration of two intravenous infusions of 300 mg ocrelizumab on day 1 and day 14, followed by 600 mg ocrelizumab every 6 months. EDTA blood samples for CyTOF analysis were obtained on the day of ocrelizumab administrations directly before the infusion at baseline (n = 31), 2 weeks (2 wk, n = 30) and 6 months (6 mo, n = 29). Cohort 2 included patients with MS in whom therapy with ocrelizumab had already been initiated before the start of this study. At the timepoint of study inclusion, patients of cohort 2 had already obtained at least 3 infusions of 600 mg ocrelizumab previously and the treatment regime was continued with intravenous infusions of 600 mg every 6 months. In analogy to cohort 1, EDTA blood samples for CyTOF analysis were obtained in cohort 2 on the day of ocrelizumab treatment, directly before the infusion 1.5 (1.5 yr, n = 50), 2.0 (2.0 yr, n = 45) and 2.5 (2.5 yr, n = 25) years after the start of ocrelizumab treatment (Figure 1A).

Additionally, differential cell counts, and serum immunoglobulin (Ig)G and IgM levels were determined at each visit before aCD20 infusions were administered. The total lymphocyte counts (Figure S4A) shown herein are from routine clinical blood tests, which enumerate B cells, T cells, and NK cells by automatic hematocytometers (Labor Berlin GmbH, Berlin, Germany). IgG and IgM were measured by immunoturbidimetry at Labor Berlin GmbH, Berlin, Germany. Plasma samples were collected at baseline and 6 months, processed, and stored at -80°C without undergoing any freeze-thaw cycles prior to analyses.

#### Ethical approval and consent to participate

The study was approved by the Ethics Committee of Charité – Universitätsmedizin Berlin (EA1/386/20) and conducted according to the Declaration of Helsinki and its later amendments. All study participants provided written informed consent before any study-related procedures were undertaken.

### Method details

#### Measurement of plasma NfL

Plasma NfL was determined by Labor Berlin GmbH Berlin, Germany, as described previously.^50^ In brief, NfL concentrations were quantified using the NF-light assay on the single molecule array (Simoa) HD-X Analyzer (Quanterix, Billerica, MA), a commercially available platform.

#### Sample processing for CyTOF based profiling

Within 1 hour after EDTA blood withdrawal, 500 μl of EDTA blood was fixed in 700 μl of Proteomic Stabilizer (Smart Tube Inc.) according to the manufacturer’s instruction and stored at -80°C until analysis by CyTOF.

#### Intracellular barcoding for mass cytometry

For CyTOF analysis, whole blood samples were thawed in Thaw/Lyse buffer and barcoded by staining with premade combinations of six different palladium isotopes: ^102^Pd, ^104^Pd, ^105^Pd, ^106^Pd, ^108^Pd and ^110^Pd (Cell-ID 20-plex Pd Barcoding Kit, Fluidigm). This multiplexing kit applies a 6-choose-3 barcoding scheme that results in 20 different combinations of three Pd isotopes. After 30 min staining at room temperature, individual samples were washed twice with cell staining buffer (0.5% bovine serum albumin in PBS, containing 2mM EDTA). Subsequently, all samples were pooled, washed and further stained with antibodies.

#### Antibodies

We used two antibody panels containing 37 antibodies each, all antibodies were validated for use in human immune cells using CyTOF, and some were additionally validated for flow cytometry.^43^^,^^51^^,^^52^ Antibody panel A targets circulating immune cells and their subsets, including T cells, granulocytes and myeloid cells, i.e., monocytes and dendritic cells (DCs), natural killer (NK) cells, activity-related markers and chemokine receptors (see Table S1 for a full list of antibodies). Antibody panel B was designed for detailed investigation of all major B cell subsets (see Table S2 for a full list of antibodies). Antibodies were purchased either pre-conjugated to metal isotopes (Standard Biotools) or from commercial suppliers in purified form and conjugated in house using the MaxPar X8 kit (Standard Biotools) according to the manufacturer’s protocol.

#### Surface and intracellular staining

Pooled barcoded samples were re-suspended in 90 μL of antibody cocktail against surface markers and incubated for 30 min at 4°C. Subsequently, cells were washed twice with cell staining buffer and incubated overnight in 2% methanol-free formaldehyde solution (FA). For intracellular staining, the stained cells were washed once with staining buffer. The samples were then stained with 100 μl antibody cocktails against intracellular molecules (see Tables S1 and S2) in permeabilization buffer for 30 min at room temperature. Afterward, cells were washed twice with staining buffer, then re-suspended in 1 mL iridium intercalator solution (Fluidigm) and incubated for 30 min at room temperature. Next, the samples were washed twice with cell staining buffer. Finally, cells were kept at 4°C in cell staining buffer until CyTOF measurement and were washed by laminar flow with MilliQ water directly prior to acquisition (Mini-1000, Curiox Biosystems). As a reference for normalization of batch effects, we included anchor samples in all CyTOF experiments. The anchor samples were prepared from whole blood samples collected from a healthy individual using the same protocol as for the patient samples.

#### Mass cytometry data processing and analysis

Boolean gating was used for de-barcoding as previously described.^51^^,^^52^ Nucleated single intact cells were manually gated according to the signals of DNA intercalators ^191^Ir/^193^Ir and event length. For de-barcoding, Boolean gating was used to deconvolute individual samples according to the barcode combination. All de-barcoded samples were then exported as individual FCS files for further analysis. Each FCS file was cleaned and compensated for signal spillover using R package CATALYST,^53^ transformed with arcsinh transformation (scale factor 5), and batch correction was implemented with a quantile normalization method to minimize batch effects^54^ prior to data analysis.

Prior to clustering analyses, CD19^+^ B cells, cPARP^-^CD66b^+^ granulocytes, cPARP^-^CD3^+^ T cells and cPARP^-^CD3^-^CD66b^-^CD14^-/+^ MNK cells were pre-gated using FlowJo (Figures S1–S3). For further clustering analysis, we used previously described scripts and workflows.^55^ For unsupervised cell population identification, we performed cell clustering with the FlowSOM^56^ and ConsensusClusterPlus^57^ packages using selected markers in each panel (Table S3). For granulocyte clustering (Panel A), we firstly identified 18 granulocyte sub-clusters based on the expression of 15 markers, including FceR1a, CD45, CD66b, CD68, CD16, CD28, CCR7, CD64, HLA-DR, CD33, CCR4, CXCR1, CXCR2, CXCR4 and CD14, then took mixed populations (cluster 17, and 18) out, and re-clustered again with 18 meta-clusters. For T cell clustering (Panel A), sub-clusters were first identified based on the expression of CD3, CD8, CD4, CCR4, CD28, CCR7, CD68, ICOS, CD161, TIGIT, CD127, CD69, CXCR4, CRTH2, CTLA4, CD45RO and HLA-DR, then one mixed population (cluster 18) was excluded, and the remaining cells were re-clustered again with 18 meta-clusters. For MNK cell clustering (Panel A), we used 18 markers, including CD68, CD11c, HLA-DR, CD14, CD64, CD33, CD47, CXCR4, CRTH2, CCR4, CCR7, CD161, CD56, TIGIT, CD8, CD141, CD16 and CD127, to identify 18 meta clusters. Then one mixed population (cluster 1) and one unidentified cluster with only HLA-DR^+^ (cluster 17) were excluded, and the remaining cells were re-clustered again with 18 meta-clusters. For B cell clustering (Panel B), we clustered B cells with 18 meta-clusters using 24 markers, including CD19, CD20, CXCR3, CD45, HLA-DR, CCR4, CD49d, CD38, IgD, IgA, IgGK, IgGL, CD95, CD27, Ki67, IgM, CD1c, CD24, Tbet, CD11c, CD123, CD62L, CD25 and CXCR4. The number of meta-clusters used for further analysis was identified based on the delta area plots (which assess the “natural” number of clusters that best fits the complexity of the data)^55^ together with visual inspection on the phenotypic heatmap with an aim to select a cluster number with consistent phenotypes that would also allow us to explore small populations. In the data analysis, we used heatmaps to visualize the scaled median expression of markers across different cell clusters. The designation of marker-negative or marker-positive populations is based on relative expression levels compared to other clusters. For dimensionality-reduction visualization we generated UMAP representations using all markers as input and down-sampled to a maximum of 1000 cells per sample.

#### NULISAseq assay

The concentrations of 250 proteins were measured in plasma samples using NULISAseq assays, which were performed at Alamar Biosciences, USA.^58^ Briefly, plasma samples stored at -80^o^C were thawed on ice and centrifuged at 10,000 x g for 10 mins. 10μl supernatant samples were then plated in 96-well plates and analyzed with Alamar’s Inflammation Panel 250 targeting mostly inflammation and immune response-related cytokines and chemokines. A Hamilton-based automation instrument was used to perform the NULISAseq workflow, starting with immunocomplex formation with DNA-barcoded capture and detection antibodies, followed by capturing and washing the immunocomplexes on paramagnetic oligo-dT beads, then releasing the immunocomplexes into a low-salt buffer, which were captured and washed on streptavidin beads. Finally, the proximal ends of the DNA strands on each immunocomplex were ligated to generate a DNA reporter molecule containing both target-specific and sample-specific barcodes. DNA reporter molecules were pooled and amplified by PCR, purified and sequenced on Illumina NextSeq 2000.

#### Data processing and normalization

For NULISAseq, sequencing data were processed using the NULISAseq algorithm (Alamar Biosciences). The sample- (SMI) and target-specific (TMI) barcodes were quantified, and up to two mismatching bases or one indel and one mismatch were allowed. Intraplate normalization was performed by dividing the target counts for each sample well by that well’s internal control counts. Interplate normalization was then performed using interplate control (IPC) normalization, wherein counts were divided by target-specific medians of the three IPC wells on that plate. Data were then rescaled, add 1 and log2 transformed to obtain NULISA Protein Quantification (NPQ) units for downstream statistical analysis.

### Quantification and statistical analysis

Statistical analysis was performed using GraphPad Prism (version 8.0.2), SPSS and R. The proportion of cell cluster across different time points within each cohort (Baseline vs 2 wk vs 6 mo in cohort 1; 1.5 yr vs 2.0 yr v 2.5 yr in cohort 2) was analyzed by a linear mixed model with random effects (Patient_id) and fixed effects (timepoint), allowing for repeated measurements and missing values. Adjusted p-values < 0.05 using the Bonferroni method were considered significant. Kruskal-Wallis test followed by Dunn’s multiple comparisons test was used to comparatively analyze marker expression across different time points within each cohort. Differential proteomic analysis was performed with a two-sided linear model t-test (Limma analysis) using the limma package. The Benjamini-Hochberg method was used to control the false discovery rate (FDR).

B cell repopulation at six months was characterized by an increased proportion of CD19^+^ cells compared to two weeks timepoint, with at least 1% of the total CD19^+^ cells detectable. Significant differences in marker expression of B12 and B14 of patients with B cell repopulation at 6 mo between baseline and 6 mo were calculated using Wilcoxon matched-pairs signed rank test and Mann–Whitney U-test (used in case of missing data (ND)). Comparison of marker expression between all patients at baseline (n=31) and patients with B cell repopulation at 1.5 yr, 2.0 yr and 2.5 yr were analyzed using Kruskal-Wallis and Dunn’s multiple comparison test.

Significance of differences in immunoglobulin level was assessed using Mann–Whitney U-test in cohort 1 (baseline vs 6 mo) and Kruskal-Wallis and Dunn’s multiple comparison test in cohort 2 (Baseline vs 1.5 yr vs 2.0 yr vs 2.5 yr). For comparison of immune cell cluster proportion between patients with and without B cell repopulation at different timepoints, two-stage step-up method of Benjamini, Krieger and Yekutieli was used to control the false discovery rate (FDR). Given the limited sample size and the exploratory nature of the study, the significance threshold was set at FDR ≤ 10% to balance type I error control with statistical power. Significance of differences in marker expression of immune cells (excluding B cells), NfL and immunoglobulin level between patients with and without B cell repopulation at different time points were analyzed using Mann–Whitney U-test. The level of significance was set at p < 0.05. Significances were presented as ∗p<0.05; ∗∗p<0.01; ∗∗∗p<0.001 and ∗∗∗∗p < 0.0001.

## Supplemental information

Document S1. Figures S1–S8

Table S1. The Panel A-antibody list, related to STAR Methods

Table S2. The Panel B-antibody list, related to STAR Methods

Table S3. Selected markers used in the clustering of T cell, granulocyte, and MNK cell-Panel A and B cell-Panel B, related to STAR Methods, and Figures 1, 3, and 4

Table S4. Patient_id of patients with B12, B14, & B16 repopulation at 6 months after treatment, related to Figure 2

Table S5. Patient_id of patients with B12, B14, & B16 repopulation at 1.5 years, 2.0 years, & 2.5 years after treatment, related to Figure 2
