# Supplementary material for: Comprehensive analysis of B cell repopulation in ocrelizumab-treated patients with multiple sclerosis by mass cytometry and proteomics
Source: iScience. 2025 Apr 8;28(5):112383. doi: 10.1016/j.isci.2025.112383 (PMC12049848; doi:10.1016/j.isci.2025.112383)
Supplement: Document S1. Figures S1–S8 [file mmc1.pdf]

iScience, Volume *28*

Supplemental information

Comprehensive analysis of B cell repopulation in ocrelizumab-treated patients with multiple sclerosis by mass cytometry and proteomics

Meng Wang, Carolin Otto, Camila Fernández Zapata, Adeline Dehlinger, Gerardina Gallaccio, Lisa-Marie Diekmann, Moritz Niederschweiberer, Patrick Schindler, Peter Körtvélyessy, Desiree Kunkel, Friedemann Paul, Klemens Ruprecht, and Chotima Böttcher

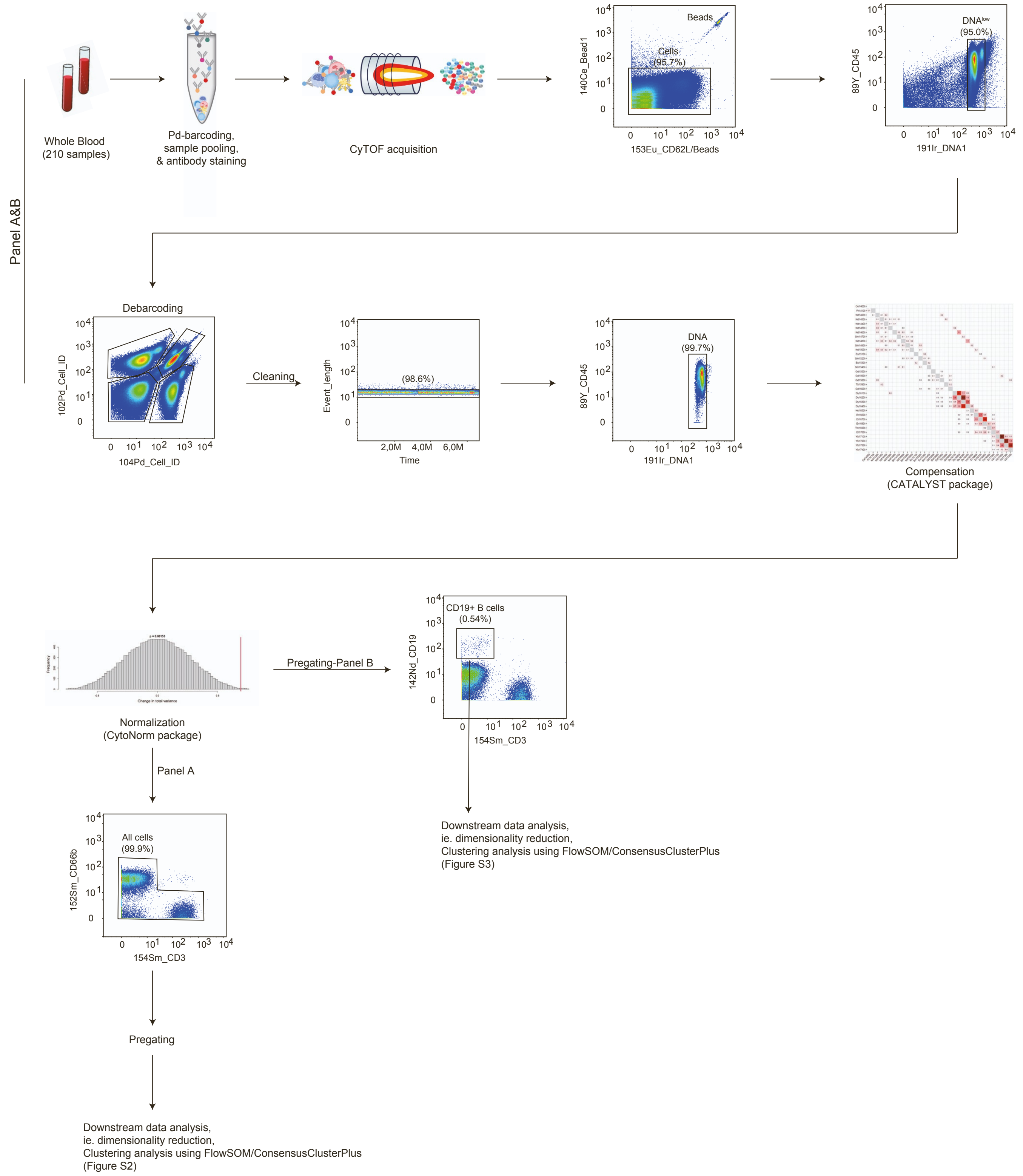

Figure S1. Schematic representation of CyTOF measurement and gating strategy from Panel A and B, related to STAR methods. A total of 210 whole blood samples were collected from MS patients. Whole blood samples were CD45-barcoded and pooled. Mixed samples were equally divided and stained with two panels (Panel A and B, Tables S1 and S2) of metal-conjugated antibodies and acquired on the CyTOF instrument. Prior to pre-gating, de-barcoding, compensation and normalization were performed. In Panel A, CD3$^+$CD66b$^+$ double positive cells were excluded from all single CD45$^+$ cells. CD66b$^+$cPARP$^-$ granulocytes, CD3$^+$cPARP$^-$ T cells and CD3$^-$CD66b$^-$cPARP$^-$ MNK cells were pre-gated and subsequently clustered (Figure S2). For Panel B, in-depth characterization of CD19$^+$cPARP$^-$ B cells (Figure S3) were performed.

A

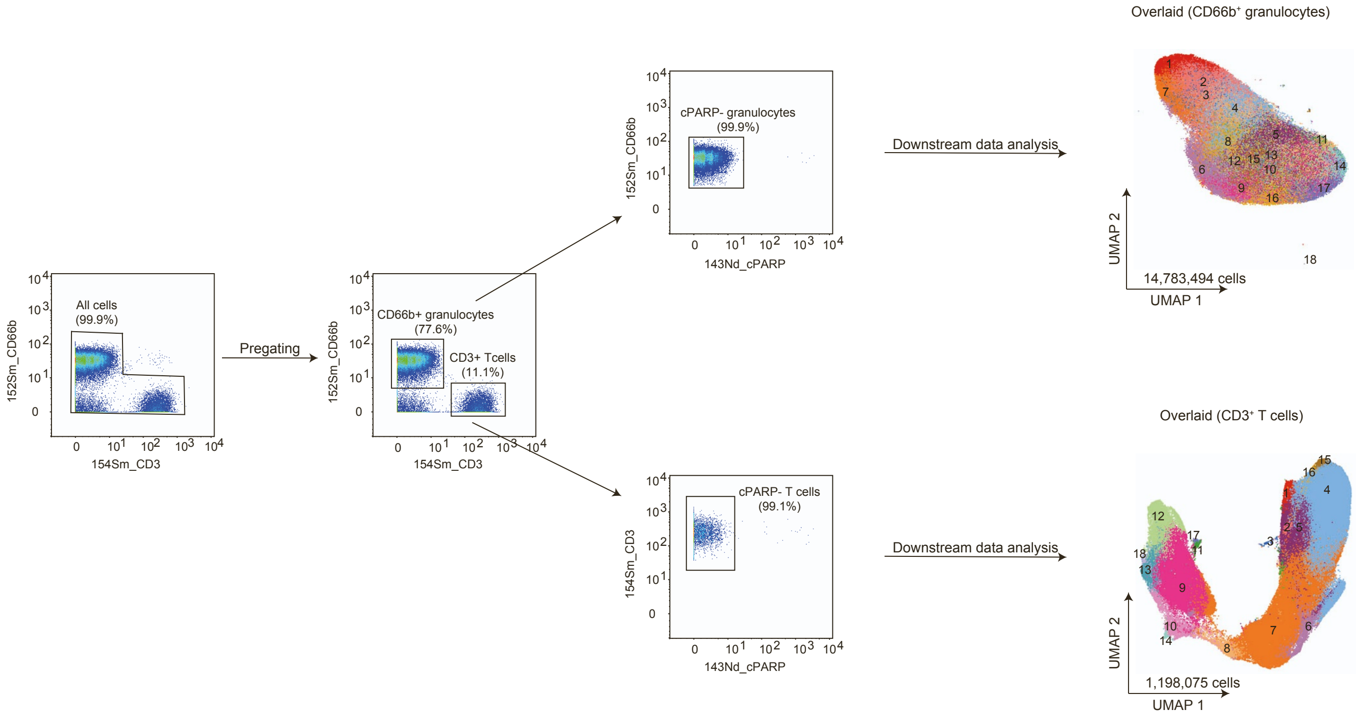

B

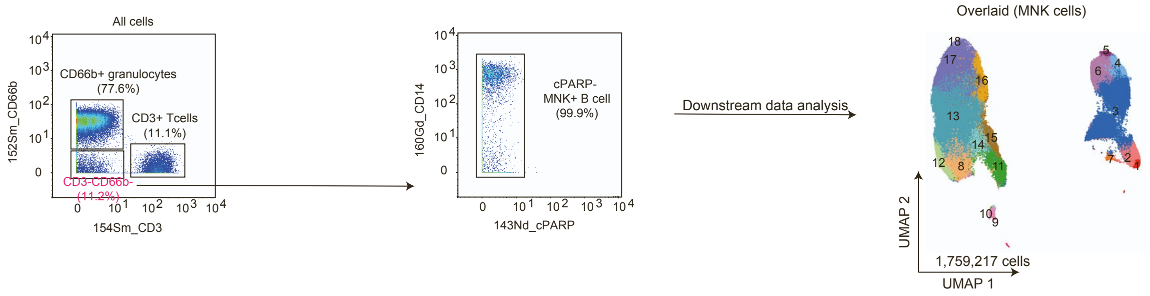

Figure S2. Gating strategy and UMAP projection of defined clusters characterized by median expression levels of selected markers for CD66b+ granulocytes and CD3$^+$ T cells (A) and MNK cells from CD3$^-$CD66b$^-$ populations (B) in Panel A (Table S1), related to **STAR methods and Figures 3 and 4**.

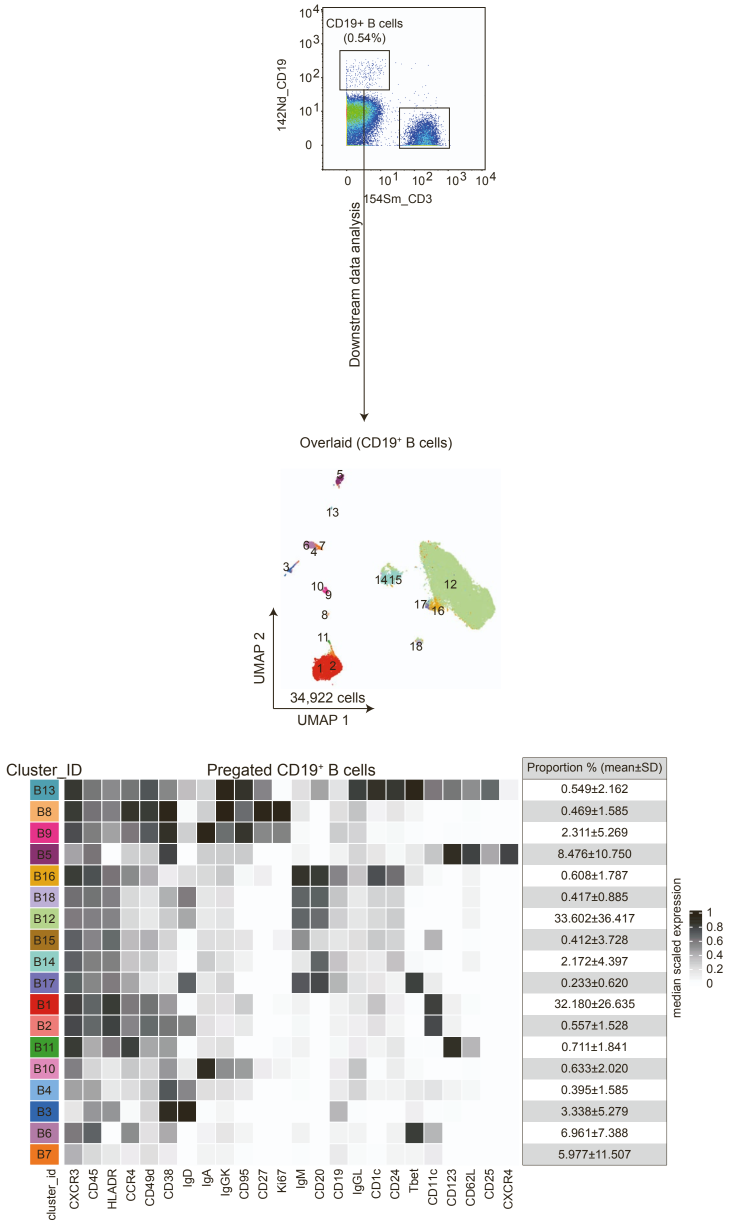

Figure S3. Gating strategy, UMAP projection and phenotypic heatmap of defined clusters based on median expression levels of selected markers for CD19⁺ B cells in Panel B (Table S2), related to **STAR methods and Figure 1**.

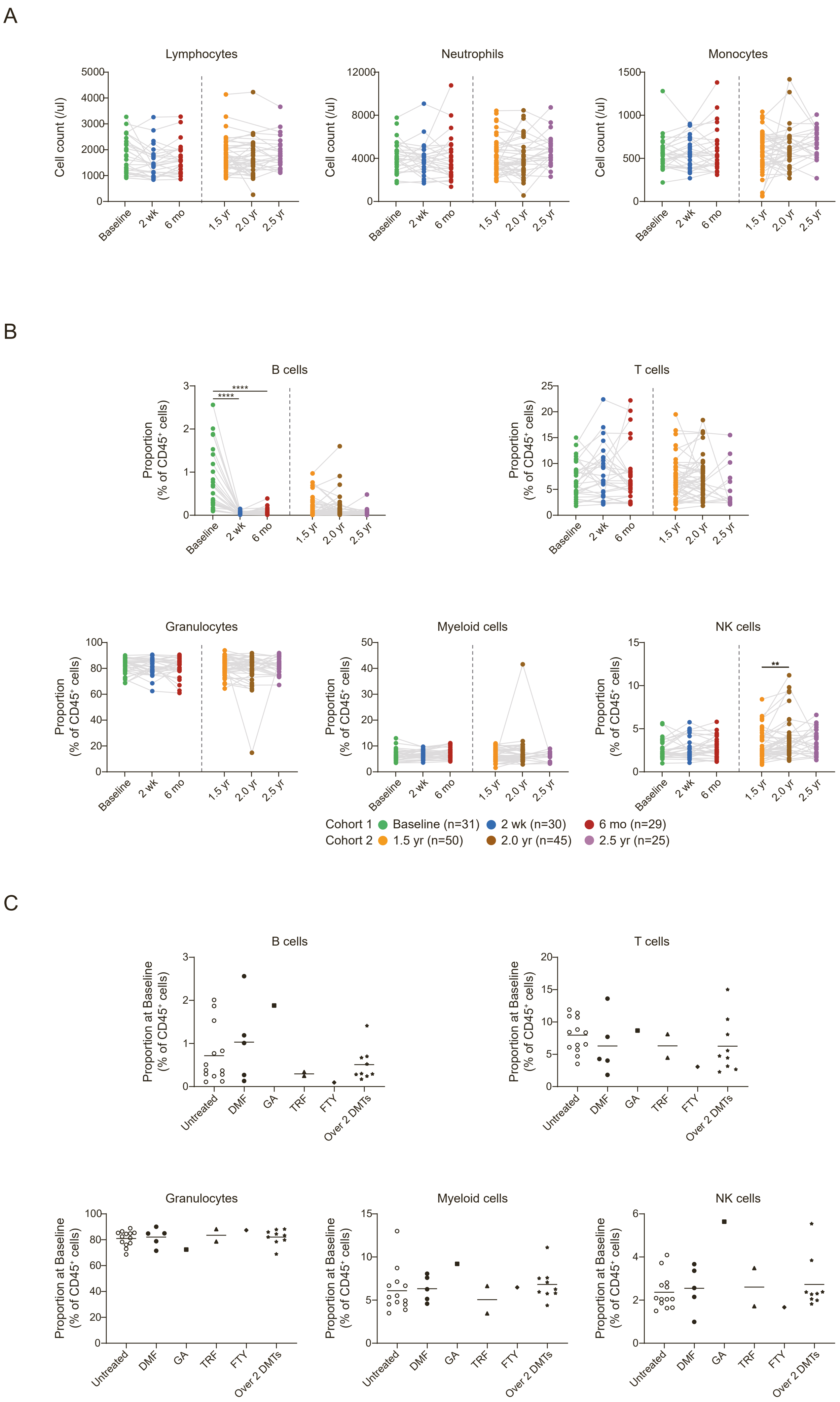
A
Lymphocytes
Neutrophils
Monocytes
Cell count (/ul)
Baseline
2 wk
6 mo
1.5 yr
2.0 yr
2.5 yr
B
B cells
T cells
Granulocytes
Myeloid cells
NK cells
Proportion (% of CD45⁺ cells)
****
**
Cohort 1 Baseline (n=31) 2 wk (n=30) 6 mo (n=29)
Cohort 2 1.5 yr (n=50) 2.0 yr (n=45) 2.5 yr (n=25)
C
Proportion at Baseline (% of CD45⁺ cells)
Untreated
DMF
GA
TRF
FTY
Over 2 DMTs

Figure S4. Compositional changes of major immune cell types after ocrelizumab treatment, related to Figure 1. (A) Absolute cell count of major immune cell types (routine blood diagnosis) in whole blood of patients from cohort 1 (Baseline (n=31), 2 wk (n=30) and 6 mo (n=29)) and cohort 2 (1.5 yr (n=50), 2.0 yr (n=45) and 2.5 yr (n=25)). Each dot represents one patient. The lines connect longitudinal data points from same patients. No statistically significant differences were detected. (B) Proportion of major immune cell types in whole blood of patients with MS from cohort 1 (Baseline (n=31), 2 wk (n=30) and 6 mo (n=29)) and cohort 2 (1.5 yr (n=50), 2.0 yr (n=45) and 2.5 yr (n=25)), determined by mass cytometry. Each dot represents one patient. The lines connect longitudinal data points from same patients. Statistical significance was determined using a linear mixed model with random effects (Patient_id) and fixed effects (timepoint). The Bonferroni method was used to control FDR. *p < 0.05, **p < 0.01, ***p < 0.001, ****p < 0.0001. (C) Proportion of major immune cell types in whole blood at baseline of MS patients with different DMTs history (Untreated, n=13; DMF, n=5; GA, n=1; TRF, n=2; FTY, n=1; Over 2 DMTs, n=9). Each dot represents one patient.

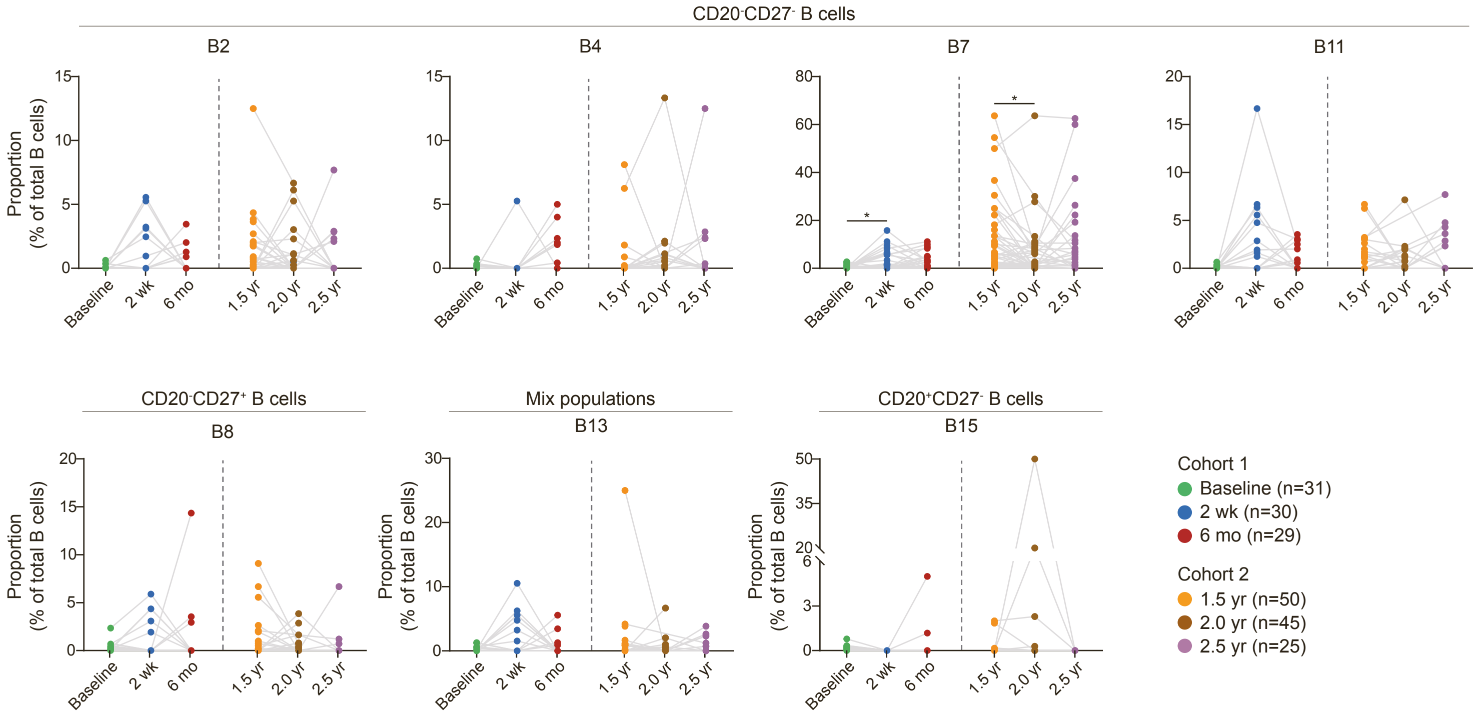

Figure S5. Proportion of seven B cell clusters (include B7 with slight change and 6 non-significant clusters) in whole blood of patients with MS from cohort 1 (Baseline (n=31), 2 wk (n=30) and 6 mo (n=29)) and cohort 2 (1.5 yr (n=50), 2.0 yr (n=45) and 2.5 yr (n=25)), related to **Figure 1**. Each dot represents one patient. The lines connect longitudinal data points from same patients. Statistical significance was determined using a linear mixed model with random effects (Patient_id) and fixed effects (timepoint). The Bonferroni method was used to control FDR. *p < 0.05, **p < 0.01, ***p < 0.001, ****p < 0.0001.

A

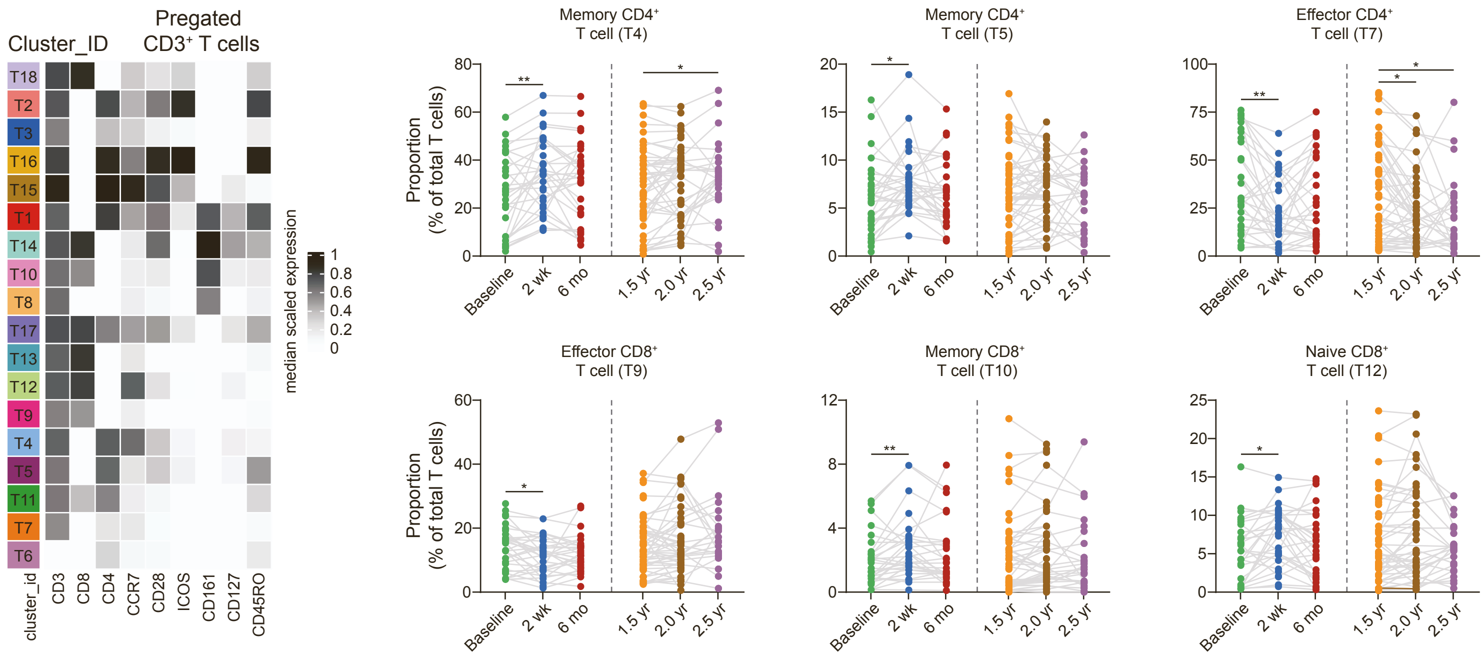

B

Cluster_id	Phenotype
M1	CD11c^{+}HLADR^{-}CD33^{+}CRTH2^{+}CD161^{lo}
M2	CD11c^{+}HLADR^{-}CD33^{+}CRTH2^{+}
N3	CD56^{-}CD16^{+}CD161^{lo}
N4	CD56^{+}CD16^{+}CD161^{hi}CD8^{+}
N5	CD56^{+}CD16^{+}CD161^{hi}CD8^{hi}
N6	CD56^{+}CD16^{+}CD161^{hi}CD8^{-}
M7	CD11c^{-}HLADR^{-}CD64^{+}CRTH2^{+}
M8	CD14^{+}CD16^{-}CCR7^{-}CD141^{-}
M9	CD11c^{-}HLADR^{+}CD141^{lo}
M10	CD11c^{+}HLADR^{hi}CD33^{+}
M11	CD14^{int}CD16^{lo}CD141^{+}
M12	CD14^{+}CD16^{-}CCR7^{lo}CD141^{-}
M13	CD14^{+}CD16^{-}CCR7^{+}CD141^{-}
M14	CD14^{+}CD16^{-}CCR7^{+}CD141^{+}
M15	CD14^{+}CD16^{lo}CD141^{hi}
M16	CD14^{hi}CD16^{-}CD64^{+}CD141^{+}
M17	CD14^{hi}CD16^{-}CD64^{+}CD141^{int}
M18	CD14^{hi}CD16^{-}CD64^{hi}CD141^{int}

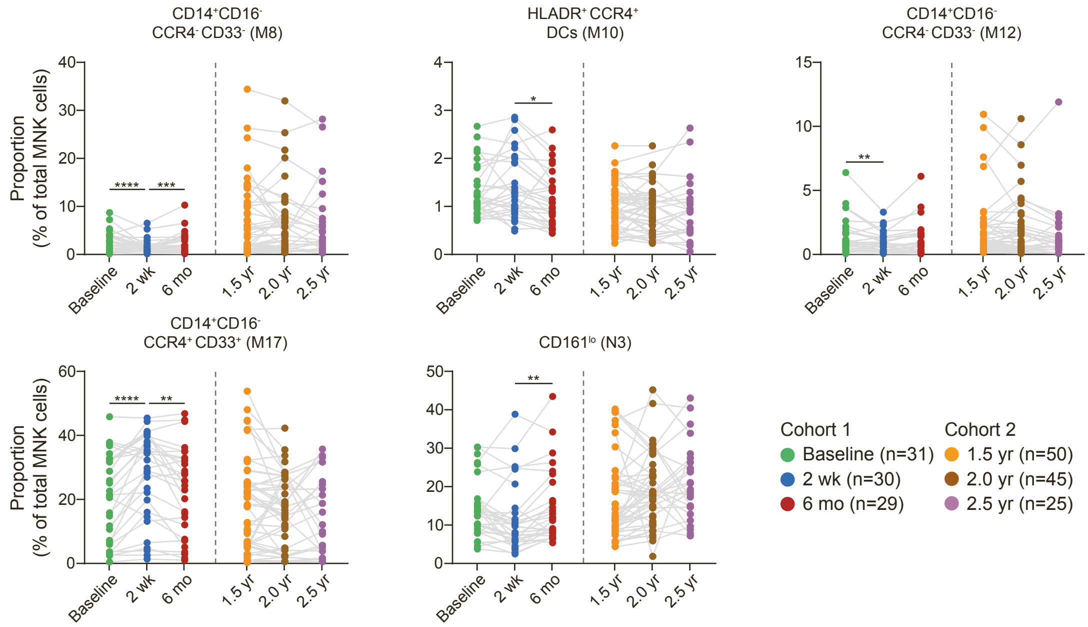

Figure S6. Compositional and phenotypic changes in T and MNK cell subsets after ocrelizumab treatment, related to Figure 1. (A) The left plot shows the phenotype of T cells determined using the FlowSOM algorithm. Box plots shows the proportion of differentially significant T cell clusters in MS patients from cohort 1 (Baseline (n=31), 2 wk (n=30) and 6 mo (n=29)) and cohort 2 (1.5 yr (n=50), 2.0 yr (n=45) and 2.5 yr (n=25)). (B) The left table shows the phenotype of MNK cells determined using the FlowSOM algorithm. Box plots shows the proportion of differentially significant MNK cell clusters in MS patients from cohort 1 (Baseline (n=31), 2 wk (n=30) and 6 mo (n=29)) and cohort 2 (1.5 yr (n=50), 2.0 yr (n=45) and 2.5 yr (n=25)). Each dot represents one patient. The lines connect longitudinal data points from same patient. Statistical significance was determined using a linear mixed model with random effects (Patient_id) and fixed effects (timepoint). The Bonferroni method was used to control FDR. *$p < 0.05$, **$p < 0.01$, ***$p < 0.001$, ****$p < 0.0001$.

Cluster_id	Phenotype
G1	$CXCR4^{-}HLADR^{int}$
G2	$CXCR4^{-}HLADR^{+}$
G3	$CXCR4^{-}HLADR^{+}CD64^{+}$
G4	$CXCR4^{-}HLADR^{+}$
G5	$CXCR4^{int}HLADR^{lo}$
G6	$CXCR4^{int}HLADR^{-}CD68^{-}$
G7	$CXCR4^{-}HLADR^{int}CD68^{-}$
G8	$CXCR4^{-}HLADR^{+}CCR7^{+}$
G9	$CXCR4^{int}HLADR^{lo}CCR7^{+}$
G10	$CXCR4^{int}HLADR^{hi}$
G11	$CXCR4^{int}HLADR^{+}CXCR1^{hi}$
G12	$CXCR4^{lo}HLADR^{+}CD14^{hi}$
G13	$CXCR4^{hi}HLADR^{lo}$
G14	$CXCR4^{lo}HLADR^{+}CCR4^{+}$
G15	$CXCR4^{+}HLADR^{+}CD64^{+}$
G16	$CXCR4^{int}HLADR^{-}$
G17	$CXCR4^{int}HLADR^{int}CCR4^{+}$
G18	$CXCR4^{-}HLADR^{hi}CD64^{+}CCR4^{+}CD14^{hi}CD68^{hi}$

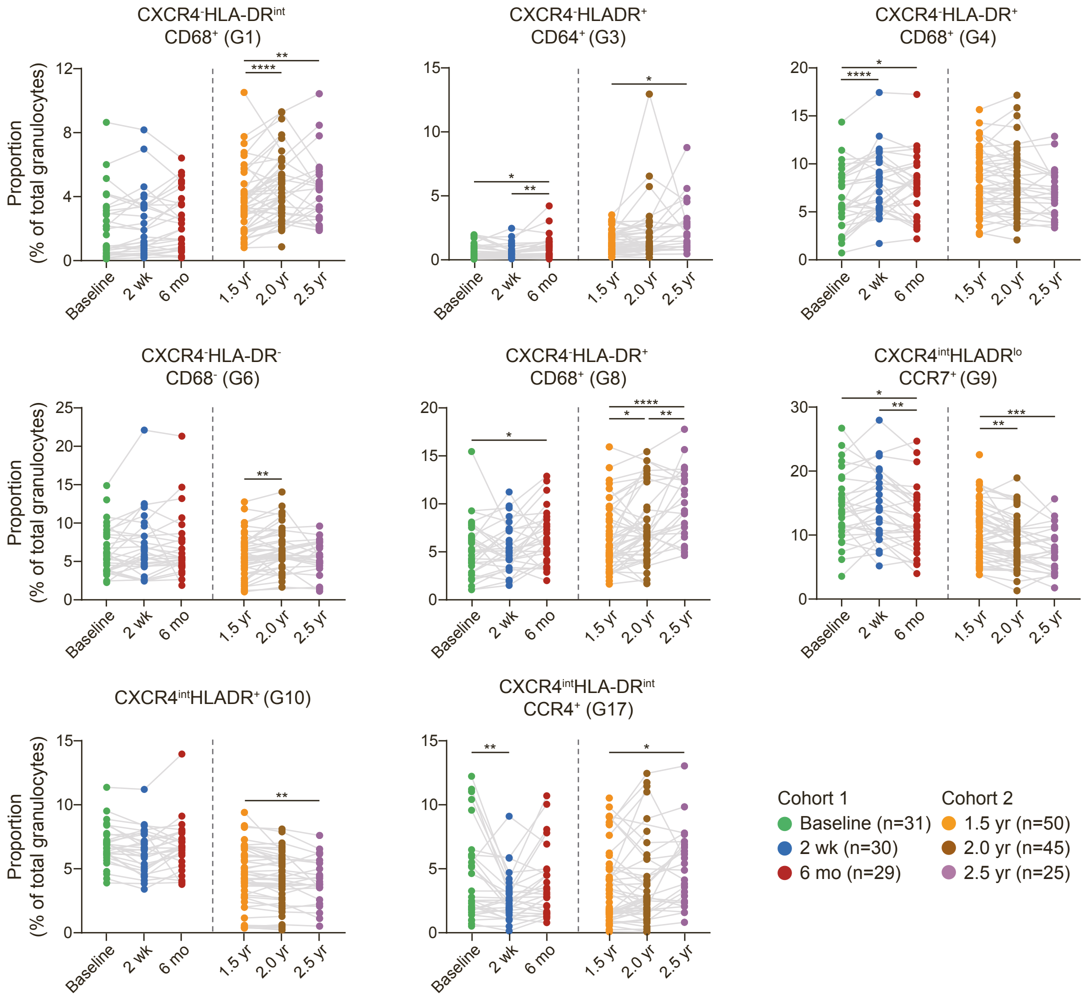

Figure S7. Compositional changes of granulocyte subsets after ocrelizumab treatment, related to Figure 1. The left table shows the phenotypes of 18 granulocyte sub-clusters determined using the FlowSOM algorithm. Box plots shows the proportion of differentially abundant granulocyte clusters in MS patients from cohort 1 (Baseline (n=31), 2 wk (n=30) and 6 mo (n=29)) and cohort 2 (1.5 yr (n=50), 2.0 yr (n=45) and 2.5 yr (n=25)). Each dot represents one patient. The lines connect longitudinal data points from the same patients. Statistical significance was determined using a linear mixed model with random effects (Patient_id) and fixed effects (timepoint). The Bonferroni method was used to control FDR. *$p < 0.05$, **$p < 0.01$, ***$p < 0.001$, ****$p < 0.0001$.

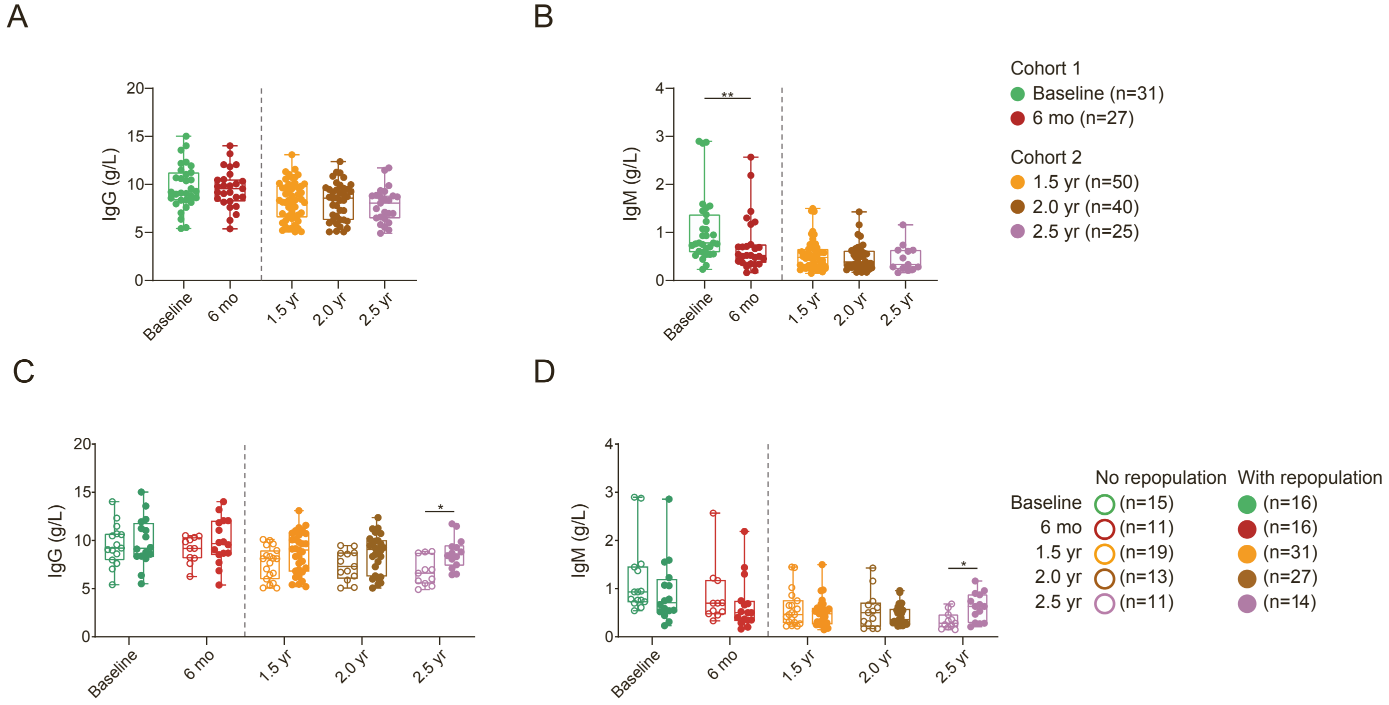

Figure S8. Changes of immunoglobulin levels after ocrelizumab treatment, related to Figure 2. (A-D) Box-plots shows levels of serum IgG (A) and serum IgM (B) of all patients and the comparison between patients with and without B cell repopulation (C and D) at five timepoints. Each dot represents one patient. Boxes extend from the 25th to 75th percentiles. Whisker plots show the min (smallest) and max (largest) values. The line in the box denotes the median. Statistical significance was determined using Wilcoxon matched-pairs rank test and Mann–Whitney U-test (cohort 1) and Kruskal-Wallis and Dunn's multiple comparison test (cohort 2). *$p < 0.05$, **$p < 0.01$, ***$p < 0.001$, ****$p < 0.0001$.
